# Machine Learning Approaches for Predicting Flow and Consistency Retention Performance in Cementitious Mixtures with Diverse Grinding Aid Dosages and Types

**DOI:** 10.3390/ma19173701

**Published:** 2026-08-31

**Authors:** Yahya Kaya, Veysel Kobya, Naz Mardani, Gulveren Tabansiz-Goc, Fatih Cavdur, Ali Mardani

**Affiliations:** 1 Department of Civil Engineering, Faculty of Engineering, Bursa Uludag University, Bursa 16059, Türkiye; 512126007@ogr.uludag.edu.tr (Y.K.); vkobya@uludag.edu.tr (V.K.); 2 Department of Mathematics Teaching, Faculty of Education, Bursa Uludag University, Bursa 16059, Türkiye; nazmardani@uludag.edu.tr; 3 Department of Industrial Engineering, Faculty of Engineering, Architecture and Design, Mudanya University, Bursa 16940, Türkiye; gulveren.tabansizgoc@mudanya.edu.tr; 4 Department of Industrial Engineering, Faculty of Engineering, Bursa Uludag University, Bursa 16059, Türkiye; fatihcavdur@uludag.edu.tr

**Keywords:** grinding aids, slump flow kinetics, machine learning, random forest, adaptive boosting, gradient boosting, multilayer perceptron, predictive analytics in construction materials

## Abstract

Grinding aids (GAs) are commonly utilized to enhance energy efficiency during clinker grinding and improve cement properties. However, alongside their benefits, GAs may lead to challenges such as reduced flow performance, loss of consistency retention, and increased demand for water or water-reducing admixtures in cementitious systems. Therefore, it is crucial to assess the flow properties and consistency retention behavior (time-dependent flow) in mixtures prepared with GAs. Given that such experiments are labor-intensive and time-consuming, predicting these performance metrics through machine learning offers substantial advantages. In this study, 29 different cements, incorporating various types and dosages of GAs, were produced. Time-dependent flow and compressive-strength tests were conducted on mixtures made with these cements. Additionally, the experimental results were compared with predictions using four machine learning models: random forest, adaptive boosting (AdaBoost), gradient boosting and multilayer perceptron. The computational results indicated that random forest outperformed the other machine learning algorithms in predicting compressive strength, whereas multilayer perceptron achieved the best overall performance for flow value prediction. Furthermore, it was concluded that random forest, adaptive boosting and gradient boosting provided acceptable performance in compressive strength prediction, while random forest and multilayer perceptron demonstrated comparatively better performance in predicting the flow value. However, the study is limited to specific types and dosages of GAs and does not include the influence of temperature, humidity, or long-term durability metrics, which may affect the generalizability of the machine learning models.

## 1. Introduction

Grinding aids (GAs) improve energy efficiency and reduce carbon emissions during clinker grinding by adsorbing onto clinker surfaces via polar functional groups (–OH, –NH_2_, –COOR, –SO_3_) [1,2,3,4]. This adsorption neutralizes surface charges, preventing crack closure, particle agglomeration, and adhesion to grinding media, thus enhancing grinding performance [3]. Beyond energy savings, GAs alter the surface characteristics and particle size distribution of cement, which can influence its compatibility with polycarboxylate-based water-reducing admixtures (PCEs) [5,6,7]. Incompatibility may cause issues such as abnormal setting, segregation, workability loss, shrinkage, or strength reduction [8,9,10,11,12,13]. Thus, evaluating cement–admixture compatibility in terms of fresh and mechanical properties is essential.

Time-dependent slump tests are commonly used to assess cement–admixture compatibility by evaluating flowability and consistency retention. Although simple in procedure, these tests become labor-intensive when applied to multiple mixtures. Similar challenges exist in preparing and curing samples for compressive strength evaluation.

Numerous machine-learning studies have been undertaken to streamline these processes [13]. Among these, we note the studies employing artificial neural networks (ANN), decision trees (DT), extra trees (ET), linear regression (LR), k-nearest neighbors (KNN), random forests (RF), support vector machines (SVM) and stochastic gradient descent (SGD) as well as boosting-based approaches, such as gradient boosting (GB), light gradient boosting (LGB) and extreme gradient boosting (XGB), for predicting some mechanical characteristics of concrete.

As detailed in the following pages, supporting the satisfactory performance of the corresponding approaches in this study, it is also observed that tree- and boosting-based approaches are also used for similar prediction purposes in recent studies. Among these, Mai et al. [14] successfully predicted the compressive strength of concrete containing ground-granulated blast-furnace slag using RF. In another study, Farooq et al. [15] used RF as well as genetic engineering programming (GEP) for compressive strength prediction. Feng et al. [16] used an adaptive boosting approach for concrete compressive strength prediction. Zeini et al. [17] predicted the strength of geopolymer-stabilized clayey soil using RF. Recently, Zhou et al. [18] used DT for the estimation of geo-polymer concrete compressive strength, whereas Mustapha et al. [19] used four different gradient boosting-based methods (i.e., gradient boosting regressor, light gradient boosting, extreme gradient boosting, and categorical gradient boosting) for the estimation of compressive strength of quaternary blend concrete.

Some studies consider using multiple machine learning approaches for similar predictions. Chou et al. [20] employed multiple aggregation regression trees (MART) and ANN methods to model concrete compressive strength. Similarly, Cheng and Cao [21] predicted concrete compressive strength using a combination of multivariate adaptive regression splines (MARS) and an ANN. Kaveh et al. [22] further extended these approaches by modeling both the compressive strength and the fresh-state properties of cementitious systems using M5 Trees and MARS methods. Marani and Nehdi [23] used various machine learning approaches, including RF, ET, GB, and XGB, to predict the compressive strength of phase change material-integrated cementitious composites. Lee et al. [24] also predicted the compressive strength of high-performance concrete using different machine-learning approaches, such as RF, AdaBoost (AB), CatBoost (CB), GB, LGB, and XGB, whereas Singh et al. [25] used DT, ET, KNN, RF, and SVM in addition to GB for compressive strength estimation of waste marble powder-incorporated concrete. More recently, Maherian et al. [26] also considered different approaches, such as ANN, LR, DT, KNN, RF, SGD, and SVM, for compressive strength estimation in nano-silica-modified concrete. In the study by Nasr et al. [27], the authors proposed an integrated approach, namely PSO-LightGBM, combining particle swarm optimization and light gradient boosting, for roller-compacted concrete containing waste ceramic aggregates exposed to freezing.

Recent studies have demonstrated the effectiveness of machine learning (ML) techniques in predicting the compressive strength of sustainable cementitious systems. Li et al. [28] developed a Gene Expression Programming (GEP) model for concrete containing NaOH-treated tire powder, outperforming traditional regression methods while reducing overfitting. Javed et al. [29] proposed hybrid ML models for steel fiber-reinforced concrete (SFRC) exposed to high temperatures, where the SVR-FFA model achieved superior accuracy (R > 0.99) and minimal prediction errors.

Ahmad et al. [30] applied ML models to forecast the compressive strength of geopolymer concrete (GPC) incorporating high-calcium fly ash. The Boosting algorithm yielded the best results (R^2^ = 0.96), with fly ash identified as the most influential input (45.3%). Similarly, Falah et al. [31] assessed deep learning neural networks (DLNN) against models such as MARS, ELM, and RF for eco-friendly concrete. DLNN exhibited the highest performance (R^2^ = 0.97, RMSE = 2.23), particularly under a 90–10% train–test split, with XGBoost highlighting cement as the most critical input variable. Tao et al. [32] also utilized XGBoost to predict the strength of FRP-confined concrete, achieving consistent accuracy (R^2^ > 0.9) and demonstrating strong feature selection capabilities.

Although machine learning (ML) models such as Random Forest, AdaBoost, Gradient Boosting, and Multilayer Perceptron have been widely used in cementitious systems, particularly for compressive strength prediction, their application to time-dependent flow behavior and consistency retention remains limited. In this study, these models are not introduced as methodological innovations but are used as predictive tools to capture complex relationships in a newly developed dataset, contributing by successfully modeling and predicting the flow value using four robust machine learning algorithms: Random Forest (RF), AdaBoost (AB), Gradient Boosting (GB), and Multilayer Perceptron (MLP). Furthermore, these same algorithms were employed to accurately predict the compressive strengths of mixtures designed to achieve specific target flow values, thereby integrating predictions of both fresh and hardened properties within a unified statistical framework.

## 2. Materials and Methods

**Figure 1:**
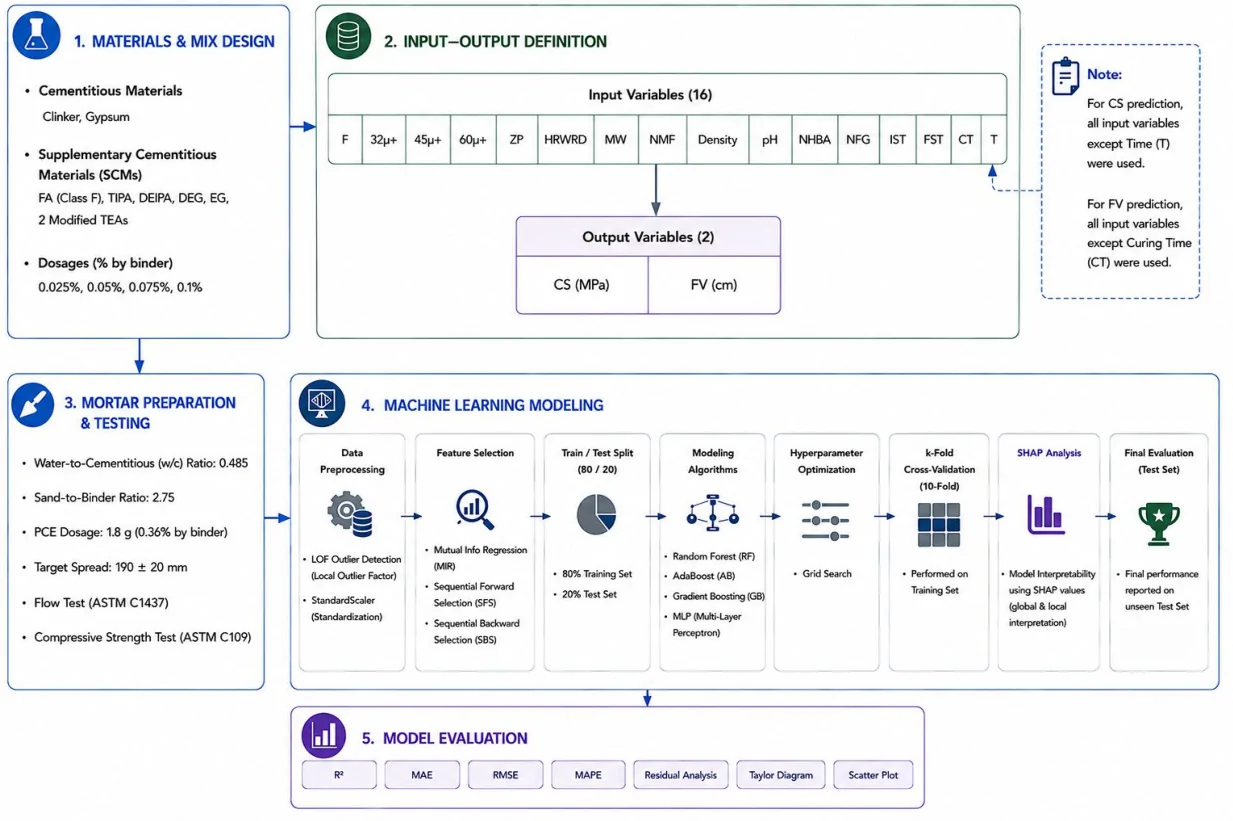
Flowchart of the experimental procedure and machine learning methods.

To provide a clear overview of the proposed framework, the study’s main stages are illustrated schematically. Figure 1 summarizes the overall methodology of the study, including the experimental procedures, dataset construction, preprocessing steps, feature selection, machine learning modeling, hyperparameter optimization, cross-validation, SHAP analysis (version 0.47.2), and model evaluation used for predicting compressive strength (CS) and flow value (FV).

### 2.1. Database Description and Preprocessing

#### 2.1.1. Materials

This study produced cements containing various types and proportions of GAs. A control cement was initially prepared by grinding 96% clinker with 4% gypsum in a laboratory-scale ball mill (MicroAnalysis Inc., 2023; Ankara, Türkiye). Alongside the control cement, seven additional GA-enhanced cements were produced by grinding with different GAs. These included commonly used commercial GAs such as triethanolamine (TEA), triisopropanolamine (TIPA), diethanolisopropanolamine (DEIPA), diethylene glycol (DEG), and ethylene glycol (EG). Additionally, two modified TEA-based GAs, synthesized through the esterification of TEA-type GA, were incorporated. All GAs were dosed at 0.025%, 0.05%, 0.075%, and 0.1% by mass of the total clinker and gypsum. These dosages were selected based on the typical effective GA range of 0.01% to 0.1% for cements with Blaine fineness between 3000 and 5500 cm^2^/g [33,34,35,36,37]. Consequently, 29 distinct CEM I 42.5R type cements conforming to the EN 197-1 Standard [38] were produced, including the control cement containing no GA. Each cement was ground to a target Blaine fineness of 4100 ± 100 cm^2^/g. Some properties of clinker and gypsum are given in Table 1.

A single type of PCE(Polisan Holding, İstanbul, Turkey) was employed to achieve the desired flow performance. The properties of the chemicals used are given in Table 2.

#### 2.1.2. Experimental Method

The influence of GAs on the flowability and compressive strength of cementitious systems was evaluated. These tests were performed according to ASTM C1437 [37] for flowability and ASTM C109 [39] for compressive strength. The water/binder ratio, sand/binder ratio, target flow value for determining admixture demand, and the amount of water-reducing admixture used in the time-dependent flow tests were kept constant at 0.485, 2.75, 190 ± 20 mm, and 1.8 g (0.36%), respectively.

The data sets obtained from the PCE requirement for target-flow and time-dependent flow experiments at constant PCE levels are presented in Table 3. The mixtures were named according to the type and dosage of GA used. For instance, mixtures produced with cement containing 0.05% DEG were labeled as 0.05 DEG. This naming convention was applied consistently across all mixtures to clearly distinguish between the different types and dosages of GAs used in the cement production process.

The produced mortar mixtures had a water-to-cement (W/C) ratio of 0.485 and a sand-to-binder ratio of 2.75. The compressive strengths were evaluated at 1, 3, 7, and 28 days using 50 mm cube specimens, following the procedures outlined in ASTM C109 [39]. The compressive strength for each mixture is the average of three individual samples, ensuring the accuracy and reliability of the results.

The data sets obtained from the compressive strength of the mixtures are presented in Table 4.

The compressive strength (CS) dataset comprised 116 observations from 29 cement formulations tested at 4 curing ages (1, 3, 7, and 28 days). Similarly, the flow value (FV) dataset comprised 145 observations from the same 29 cement formulations evaluated at 5 elapsed times (0, 15, 30, 45, and 60 min).

**Figure 2:**
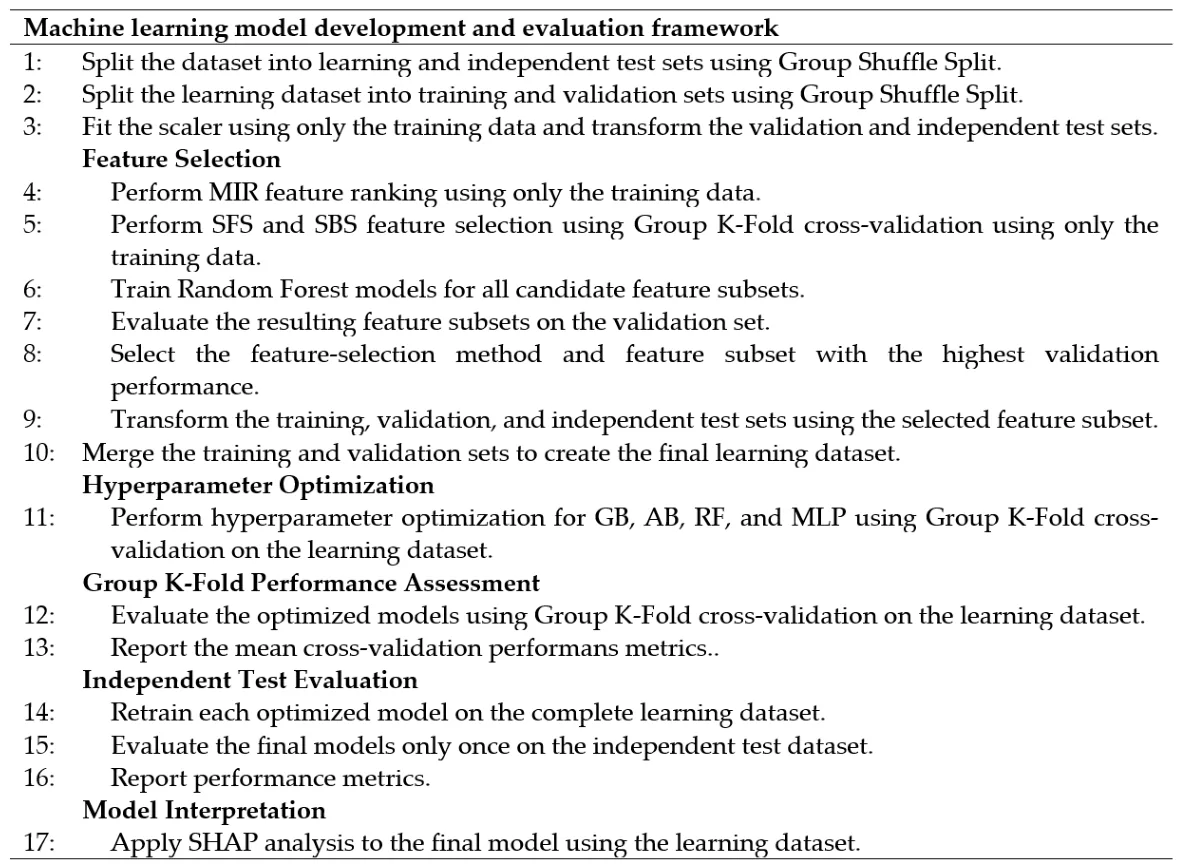
Machine learning model development framework.

Figure 2 presents the machine learning model development and evaluation framework adopted in this study. Initially, the dataset was divided into learning and independent test datasets using the Group Shuffle Split strategy. The learning dataset was subsequently partitioned into training and validation datasets for feature selection and model development. Following data preprocessing and feature selection, the training and validation datasets were merged to form the final learning dataset. Hyperparameter optimization and Group K-Fold cross-validation were then performed on the learning dataset. Finally, the optimized models were evaluated on the independent test dataset, and SHAP analysis was conducted to improve model interpretability.

**Figure 3:**
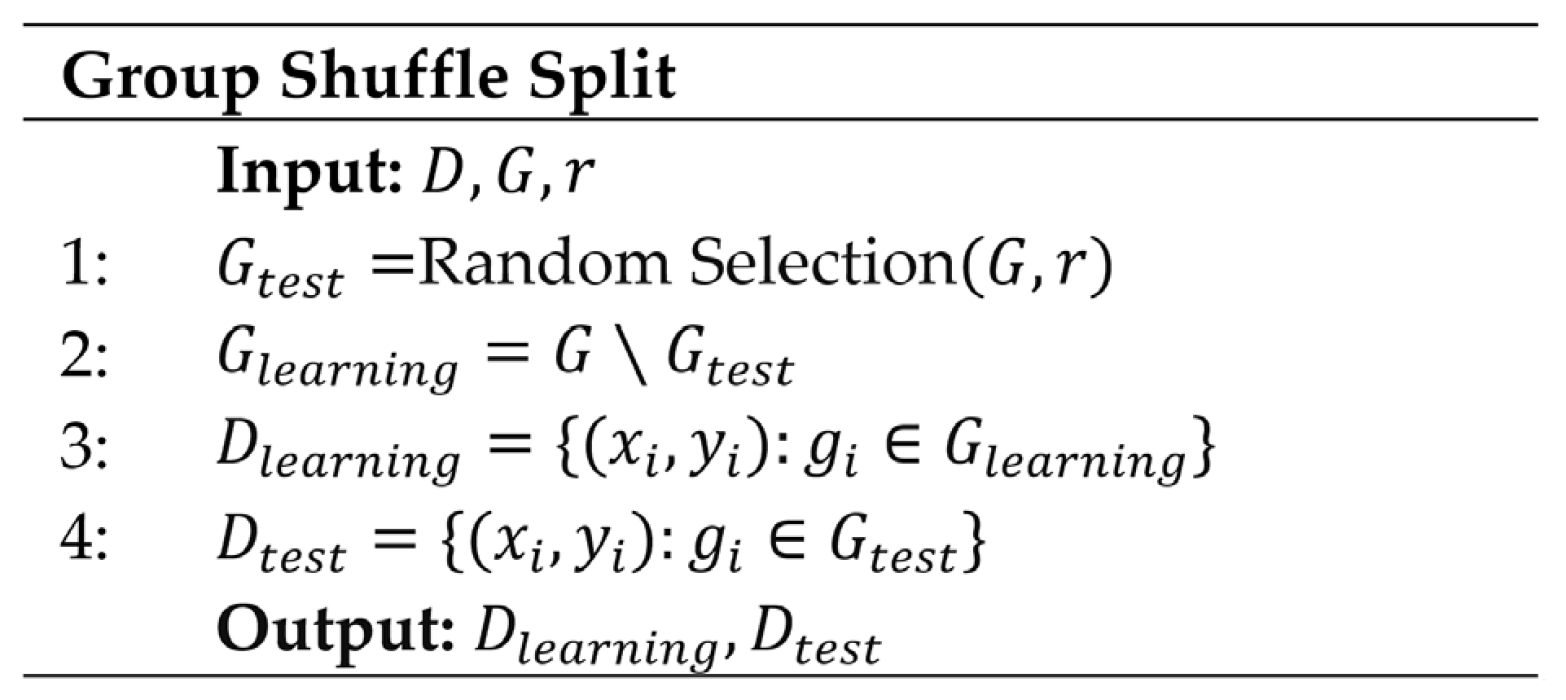
Group Shuffle Split pseudocode.

**Figure 4:**
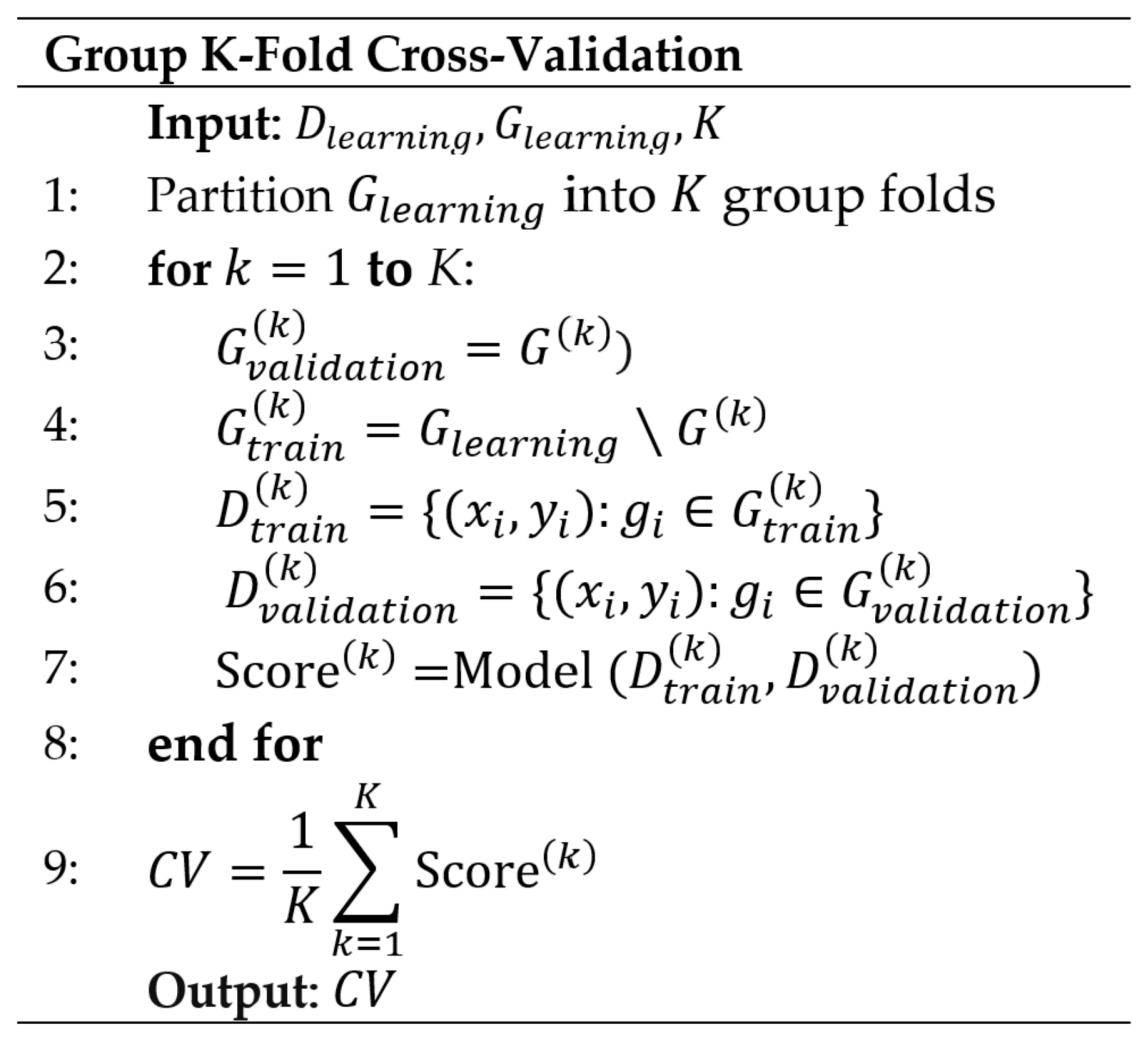
Group K-Fold Cross-Validation pseudocode.

The group-based data partitioning and validation procedures adopted in this study are summarized in Figure 3 (Group Shuffle Split) and Figure 4 (Group K-Fold Cross-Validation). Figure 3 presents the Group Shuffle Split procedure used to generate the learning and independent test datasets while preserving formulation groups. Figure 4 illustrates the Group K-Fold cross-validation procedure employed during feature selection, hyperparameter optimization, and model performance assessment.

To further support the data preprocessing procedure, a multivariate outlier analysis was also performed in this study. In this context, the Local Outlier Factor (LOF) method was applied to evaluate observations based on local density. The analysis results indicated that no anomalous observations or significant multivariate outliers were detected in the dataset. In addition, univariate outlier analyses were conducted for each variable prior to model training, and the dataset was carefully examined through both visual and statistical inspections. These findings demonstrated that the dataset had a consistent structure and was suitable for modeling.

Table 5 summarizes the statistics for the input and outcome variables, including mean, standard error, median, mode, standard deviation, range, minimum, and maximum for each variable. These statistical parameters were presented to evaluate the overall structure, distribution characteristics, and variability of the dataset prior to the modeling stage. For example, the compressive strength (CS) values ranged from 13.17 MPa to 64.85 MPa with a standard deviation of 15.75 MPa, indicating a broad distribution of mechanical performance levels within the dataset. Similarly, the flow value (FV) ranged from 14 to 21.2 cm, indicating sufficient variability in the fresh-state properties. In the dataset, input attributes other than Time (T) were used for CS prediction, and input attributes other than CT (Curing Time) were used for FV prediction.

The dataset was first divided into learning and independent test datasets using the Group Shuffle Split strategy. Subsequently, the learning dataset was further partitioned into training and validation datasets using another Group Shuffle Split procedure while preserving formulation-based grouping. The standard scaler was then fitted exclusively using the training dataset. The scaling parameters obtained from the training data were subsequently applied to the validation and independent test datasets using only the transform operation. Therefore, no information from the validation or independent test datasets was used during the scaling process.

The dataset was split into 80% for training and 20% for testing. The independent test set consisted of the formulations 0.1 TIPA, 0.05 DEIPA, 0.025 DEG, 0.1 M-TEA-1, 0.05 M-TEA-2, and 0.1 M-TEA-2, while the remaining formulations were used in the learning dataset for training and validation. Formulation selection for the independent test set was performed completely at random using the Group Shuffle Split strategy, and only random_state = 42 was specified to ensure reproducibility of the data partitioning.

#### 2.1.3. Feature Selection

Feature selection methods offer several advantages, such as reducing data collection costs and making classification models more straightforward to understand [41]. These methods can be categorized into filtering, wrapping, or embedding approaches [42,43]. The advantages and disadvantages of these techniques have been summarized by Ladha and Deepa [44], Saeys et al. [45] and Bolon-Canedo et al. [46]. In this study, three feature selection methods are used: Mutual Info Regression (MIR), Sequential Forward Selection (SFS), and Sequential Backward Selection (SBS).

Initially, the MIR method evaluates the dependability of original attributes by measuring the mutual information between two variables utilizing probability density functions p(x), p(y), and p(x,y). In this method, picking features with the highest mutual information is essential. Typically, the top m individual features are chosen during a sequential search. The SFS method is a wrapper-based feature selection approach that starts with an empty feature set and sequentially adds the feature that provides the greatest improvement in model performance at each iteration. The process continues until the addition of new features no longer provides a significant performance improvement. Unlike the SFS method, the SBS method operates inversely, starting with the complete features and progressively discarding those deemed unnecessary. The SBS method is notably efficient in extensive feature set scenarios [44].

To prevent data leakage, formulation IDs were defined as grouping variables throughout all data partitioning procedures. Accordingly, the dataset was divided into learning and independent test datasets using the Group Shuffle Split method, and the learning dataset was subsequently partitioned into training and validation datasets. MIR, SFS, and SBS were applied exclusively to the training dataset. During the SFS and SBS procedures, Random Forest was used as the evaluation model, and Group K-Fold cross-validation was employed as the evaluation criterion. The resulting feature subsets were then evaluated on the validation dataset, and the feature subset providing the highest validation performance was selected. The overall machine learning framework, Group Shuffle Split strategy, and Group K-Fold cross-validation procedure are presented in Figure 2 and Figure 3, and Figure 4, respectively.

In the MIR approach, Fineness, 32μ+, 45μ+, 60μ+, ZP, HRWRD, MW, and NMF were removed from the analysis. In the SBS approach, Fineness, 32μ+, ZP, HRWRD, Density, pH, NHBA, IST, and CT were excluded. Similarly, the SFS method removed Fineness, 32μ+, ZP, Density, pH, and MW features from the analysis. Consequently, Density, pH, NHBA, NFG, IST, FST, and CT were selected as the final input variables for CS estimation according to the MIR results, which achieved the highest validation performance using the Random Forest model.

In the MIR approach, only Density was removed from the analysis. Similarly, the SFS method excluded Density and IST features, while the SBS method removed Density, IST, and FST from the analysis. Consequently, Fineness, 32μ+, 45μ+, 60μ+, ZP, HRWRD, MW, NMF, pH, NHBA, NFG, and Time were selected as the final input variables for FV estimation according to the SBS results, which achieved the highest validation performance using the Random Forest model.

### 2.2. Hyperparameter Tuning and Optimization

Hyperparameters are crucial in optimizing a model’s learning process within supervised machine learning, including regression and classification models. These parameters are vital for a model’s training phase, as Yang and Shami [47] noted. To pinpoint the most suitable values for these parameters, one might rely on the machine learning package’s default configurations or embark on a process of trial and error. However, as Singh et al. [48] point out, this trial-and-error method can be exceedingly time-consuming and labor-intensive. To circumvent these challenges, employing hyperparameter optimization and tuning strategies can be a more efficient way to allocate time and resources.

Selecting the correct hyperparameters for a model is crucial as these parameters aim to ensure minimal error and maximum accuracy in the model’s performance. The Grid search method has been extensively utilized to assess the effectiveness of ML models under various hyperparameter configurations [49]. Cross-validation ensures the model’s reliability and capability to generalize well to new data, thus measuring the model’s effectiveness. Specific hyperparameters and their potential ranges are identified for every machine-learning approach. These models are then assessed by applying the Grid search method to these hyperparameters, followed by a 10-fold Group K-fold cross-validation process on the learning dataset.

The dataset used for model development consisted of repeated observations obtained from distinct cement formulations. Therefore, formulation IDs were defined as grouping variables to prevent potential data leakage during model development and evaluation. After the optimal feature subset had been selected, the training and validation datasets were merged to create the learning dataset. Hyperparameter optimization and model performance assessment were then performed using 10-fold Group K-Fold cross-validation on the learning dataset.

Throughout this process, the mean absolute error (MAE), root mean squared error (RMSE), mean absolute percentage error (MAPE), and coefficient of determination (R^2^) were calculated for each fold, and the average values across all ten folds were subsequently reported. The hyperparameter search ranges considered for each machine learning model, together with the corresponding optimal hyperparameter configurations identified for CS and FV prediction models, are presented in Table 6 and Table 7, respectively. The corresponding 10-fold Group K-Fold validation results obtained using the optimal hyperparameter settings are reported in Table 8 and Table 9 for CS and FV prediction models, respectively.

### 2.3. Description of Methods Used

In this study, four machine learning algorithms—Gradient Boosting (GB), AdaBoost (AB), Random Forest (RF), and Multilayer Perceptron (MLP)—were selected considering the characteristics of the dataset and the need to capture complex relationships between the input features and target variables. GB was chosen for its proven high accuracy and robustness, especially with relatively small datasets. AB and RF were included due to their strengths in ensemble learning and handling nonlinearities, while MLP was selected as a representative neural network model capable of modeling complex patterns.

At the preliminary stage, other algorithms were also evaluated, but they did not provide significant improvements over these four models. Additionally, increasing the number of models would have complicated the analysis and reduced the interpretability of the results. Therefore, to ensure clarity and focus on the most promising approaches, the study concentrated on these four well-performing models.

#### 2.3.1. Random Forest Regressor

The Random Forest method is recognized for its effectiveness in generating regression trees with reduced variance, highlighting its status as a potent method within ensemble learning [50]. This technique constructs an array of decision trees, each trained on a different bootstrapped portion of the dataset (sampled with replacement), and considers a distinct subset of features at every split. Such a methodology promotes regularization naturally, fosters diversity across the trees, and aids in averting overfitting efficiently, thereby enhancing the model’s predictive performance. The combined prediction typically results from averaging (or sometimes, using a weighted average of) the predictions from each tree. This approach is particularly adept at navigating the intricacies of complex data relationships. For a reliable assessment of the model’s performance and to preclude an overly optimistic evaluation, the implementation of k-fold cross-validation is commonly recommended.

#### 2.3.2. Adaboost Regressor

The AdaBoost method stands out as an ensemble method in machine learning, particularly for tackling regression challenges. This technique is marked by its sequential process of refining predictions through iterative adjustments [50]. Diverging from methods like GB, it assigns weights to training samples based on their previous iteration performance, specifically highlighting incorrectly classified samples. This strategy enables subsequent models to concentrate on the more complex predictions. The collective prediction results from aggregating the models’ weighted predictions, with a preference for those models that excel in managing challenging scenarios. The learning rate in AB is crucial, as it determines how the weights of each model are adjusted, impacting their overall contribution to the ensemble’s final prediction [51]. To validate its predictive strength and prevent overfitting, AB typically employs k-fold cross-validation.

#### 2.3.3. Gradient Boosting Regressor

The Gradient boosting method, a technique within the ensemble learning framework, is adept at addressing regression challenges, as noted by Nguyen et al. [52]. This approach enhances prediction accuracy by sequentially combining the efforts of multiple basic models. Each cycle aims to close the gap between the actual outcomes and the combined ensemble forecast by guiding each new model to correct the loss function’s negative gradient based on the ensemble’s current predictions. Summing up the contributions of all models yields the ultimate prediction. A crucial parameter in this technique is the learning rate, which dictates the magnitude of adjustment each basic model undergoes. A lower learning rate ensures smoother convergence and enables the detection of intricate patterns within the regression data, a point underscored by Touzani et al. [53]. K-fold cross-validation is typically employed to assess a model’s efficacy and generalizability. This process involves dividing the data into k segments, then training and testing the model k times to guarantee thorough evaluation and reliability, with the validation results being documented after each iteration. In this study, the number of folds was set to ten. Due to its proficiency in handling complex data relationships and reducing error margins, GB is a valuable tool in machine learning across various applications.

#### 2.3.4. Multilayer Perceptron Regressor

The Multilayer Perceptron (MLP) is a fundamental type of ANN that is widely used in various machine learning tasks, including both regression and classification [54]. The concept of ANN, first introduced by McCulloch and Pitts [55], has become one of the cornerstones of modern deep learning. MLPs are feed-forward networks composed of an input layer, one or more hidden layers, and an output layer. Each neuron in a layer is fully connected to all neurons in the next layer, and the number of neurons in the input layer corresponds to the number of features in the dataset. The number of hidden layers and neurons per layer may vary depending on the specific problem [56]. Thanks to their flexibility in activation functions, MLPs can effectively model non-linear relationships using functions such as sigmoid, tanh, or ReLU.

MLPs are trained through a learning process involving both forward propagation and backpropagation algorithms. The difference between the predicted output and the actual value is computed using a loss function; this error is then propagated backward through the network, and the weights are updated using optimization algorithms. One of the most commonly used algorithms is Stochastic Gradient Descent (SGD), which updates model parameters using small subsets of data in each iteration. A more advanced method, Adam, employs adaptive learning rates and gradient estimates to enable faster and more stable convergence. In addition, L-BFGS (Limited-memory Broyden–Fletcher–Goldfarb–Shanno), which utilizes second-order derivative information, provides highly accurate optimization, particularly for small datasets. With their layered and flexible architecture, MLPs remain a powerful and versatile solution in modern machine learning applications [57].

### 2.4. Performance Evaluation of Models

The assessment of the machine learning models’ effectiveness was conducted using statistical measures such as R^2^, MAE, RMSE, and MAPE. The *R*^2^ value measures the model’s precision, evaluating the discrepancy between the model’s predictions and the actual data points. A higher *R*^2^ value indicates less bias, approaching one, while a lower value, closer to zero, suggests a more significant bias. Evaluating the accuracy of these machine learning models involved using Equations (1)–(4), where *n* represents the dataset size, $P_{i}$ denotes the predicted results, and $E_{i}$ represents the observed outcomes.

(1) $$R^{2}=1-\frac{\sum_{i=1}^{n}(E_{i}-P_{i}{)}^{2}}{\sum_{i=1}^{n}(E_{i}-\bar{E}{)}^{2}}$$

(2) $$MAE=\frac{1}{n}\sum_{i=1}^{n}\left|P_{i}-E_{i}\right|$$

(3) $$RMSE=\sqrt{\sum_{i=1}^{n}\frac{{\left(P_{i}-E_{i}\right)}^{2}}{n}}$$

(4) $$MAPE=\frac{100\%}{n}\sum_{i=1}^{n}\frac{\left|P_{i}-E_{i}\right|}{E_{i}}$$

### 2.5. SHAP Analysis

SHAP (SHapley Additive exPlanations) is an interpretability method based on cooperative game theory, using Shapley values to quantify the contribution of each input feature to a model’s prediction [58,59]. In this framework, features are considered players, and the model output is the game’s result [60].

Originally proposed by Lundberg and Lee [61], SHAP belongs to the class of additive feature attribution methods. TreeSHAP, its optimized version for tree-based models like DT, RF, and XGBoost, enables fast and consistent computation of feature contributions. While TreeSHAP is specifically designed for tree-based algorithms, KernelSHAP provides a model-agnostic alternative that can be applied to non-tree-based models, including MLP networks. SHAP effectively addresses the black-box nature of machine learning models, offering clear insights into how predictions are made [62,63].

Additionally, SHAP provides visual tools such as summary and dependence plots to illustrate both the direction and magnitude of each feature’s influence [64,65].

## 3. Results and Analysis

### 3.1. Experimental Results

#### 3.1.1. Compressive Strength Results

The 1-day compressive strengths of mixes incorporating 0.025% TEA showed no notable deviation from the control sample. However, enhancements of 10%, 12%, and 20% were recorded for formulations with 0.05%, 0.075%, and 0.1% TEA, respectively. These improvements are attributed to TEA’s role in promoting early hydration of the C_3_A phase [38,66], with the effect becoming more pronounced as dosage increases. By the third day, this early strength advantage had largely diminished, likely due to a retardation in the formation of C_3_S and C_2_S phases—a consequence of accelerated C_3_A hydration [66]. At 28 days, strength gains reached up to 20% over the control.

In TIPA-containing systems, the 1-day strength gains were more modest, with increases of 5% and 9% at 0.1% and 0.05%, respectively. These outcomes are linked to TIPA’s influence on enhancing the hydration of the C_4_AF phase, which contributes to progressive strength development over time [34,38,40,66]. No major differences were seen at 3 days, but by 28 days, compressive strength improvements of up to 25% were observed.

Conversely, DEIPA resulted in a 1-day reduction in strength of 11–15% relative to the control. DEIPA’s stronger interaction with both C_3_A and C_4_AF phases leads to accelerated hydration of these minerals [66], which may delay C-S-H formation, thereby lowering early strength. This drawback was no longer evident at 3 days, and at 28 days, DEIPA-containing mixes showed strength increases of 17–23%.

When comparing glycol-based admixtures, ethylene glycol (EG) demonstrated superior performance over diethylene glycol (DEG). However, consistent with earlier studies [2,36], amine-based additives generally outperformed their glycol-based counterparts in enhancing compressive strength.

M-TEA formulations produced early-strength levels comparable to those of standard TEA mixes but exhibited significantly greater strength at 28 days, indicating the effectiveness of the modification process in improving long-term mechanical performance.

#### 3.1.2. Time-Dependent Flowing Results

To evaluate the time-dependent flowing behavior of the mixes under identical conditions, mortar samples were prepared using a fixed PCE dosage of 0.36%. Under these conditions, the control mix exhibited the highest flow compared to the others. An overall decline in flow performance was observed with increasing GA dosage, regardless of the GA type. According to Sun et al. [40], this trend may be attributed to the preferential adsorption of PCE on larger cement particles, leading to higher initial flowability in cements with coarser grains. Conversely, when the cement has a narrower particle size distribution but maintains the same Blaine fineness, the mixture tends to be more cohesive, which can hinder flow. This is likely due to the increased presence of fine particles, which elevate water demand and thus reduce flow.

Independent of GA type and dosage, GA-containing mixtures demonstrated superior consistency retention compared to the control mix. Sun et al. [40] suggested that a finer particle size distribution enhances the gradual adsorption of PCE over time. The improved consistency retention observed in GA-containing cements can be attributed to both a lower initial adsorption of PCE—resulting in a greater quantity of free PCE in the system—and the beneficial influence of fine particles on delayed adsorption kinetics. Among the GAs tested, TIPA delivered the highest consistency retention, exceeding the control by 6%, while M-TEA-2 recorded the lowest performance, still outperforming the control by 2%.

All modeling and analysis procedures in this study were carried out using Python (version 3.12.7). The analyses were conducted using the latest stable version of Python together with up-to-date versions of the relevant libraries. The scikit-learn (version 1.4.2) library was utilized for feature selection, data preprocessing, model development, hyperparameter optimization, and performance evaluation through modules including feature_selection, ensemble, metrics, model_selection, GridSearchCV, neural_network, GroupShuffleSplit, and GroupKFold. In addition, the SHAP library was employed for model interpretability and feature contribution analyses. TreeSHAP was used for tree-based models, whereas KernelSHAP was employed for the MLP model.

The prediction performances presented in the following sections were obtained using an independent test dataset. To prevent potential data leakage, feature selection, hyperparameter optimization, and model development were performed exclusively on the learning dataset. After the optimal feature subset and hyperparameter configurations had been identified, each model was retrained using the complete learning dataset and subsequently evaluated only once on the independent test dataset. Therefore, the reported results represent the generalization capability of the developed models on previously unseen cement formulations.

### 3.2. Compressive Strength Prediction

#### 3.2.1. Random Forest Model

**Figure 5:**
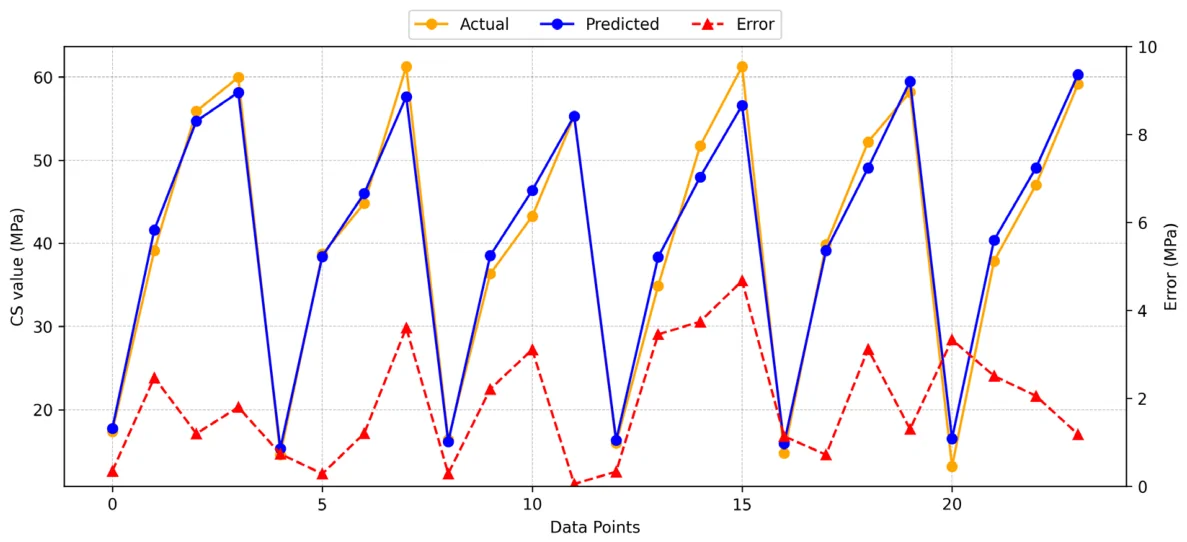
RF model, model estimation, and distribution of accurate and absolute error values for CS.

Figure 5 presents the results from applying the RF model to predict CS. Among the evaluated models, the RF model achieved the best overall predictive performance and exhibited relatively low deviations between the experimental and predicted values. The obtained R^2^ score of 0.9809 indicates a strong agreement between the actual and predicted results. Figure 5 illustrates the distributions of predicted, actual, and absolute error values for the RF model. The maximum observed error was 4.67 MPa, while the average error value was 1.87 MPa. The error distribution demonstrates that the RF model provides reliable and accurate predictions for CS estimation.

#### 3.2.2. Adaboost Model

**Figure 6:**
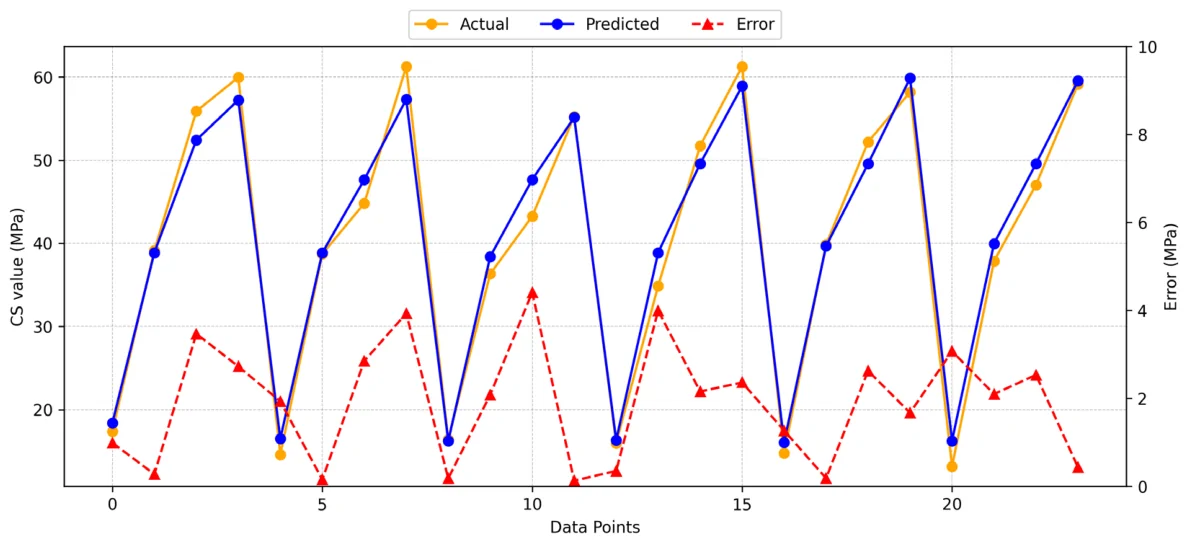
AB model, model prediction, and distribution of true and absolute error values for CS.

Figure 6 presents the results obtained from the AB model for predicting CS. The AB model produced accurate predictions with relatively small deviations between the experimental and predicted values. The obtained R^2^ score of 0.9802 indicates a high prediction capability. Figure 6 shows the distributions of predicted, actual, and absolute error values for the AB model. The highest observed error value was 4.41 MPa, while the average error value was 1.91 MPa. Although the AB model demonstrated highly competitive performance, its overall accuracy remained slightly lower than that of the RF model.

#### 3.2.3. Gradient Boosting Model

**Figure 7:**
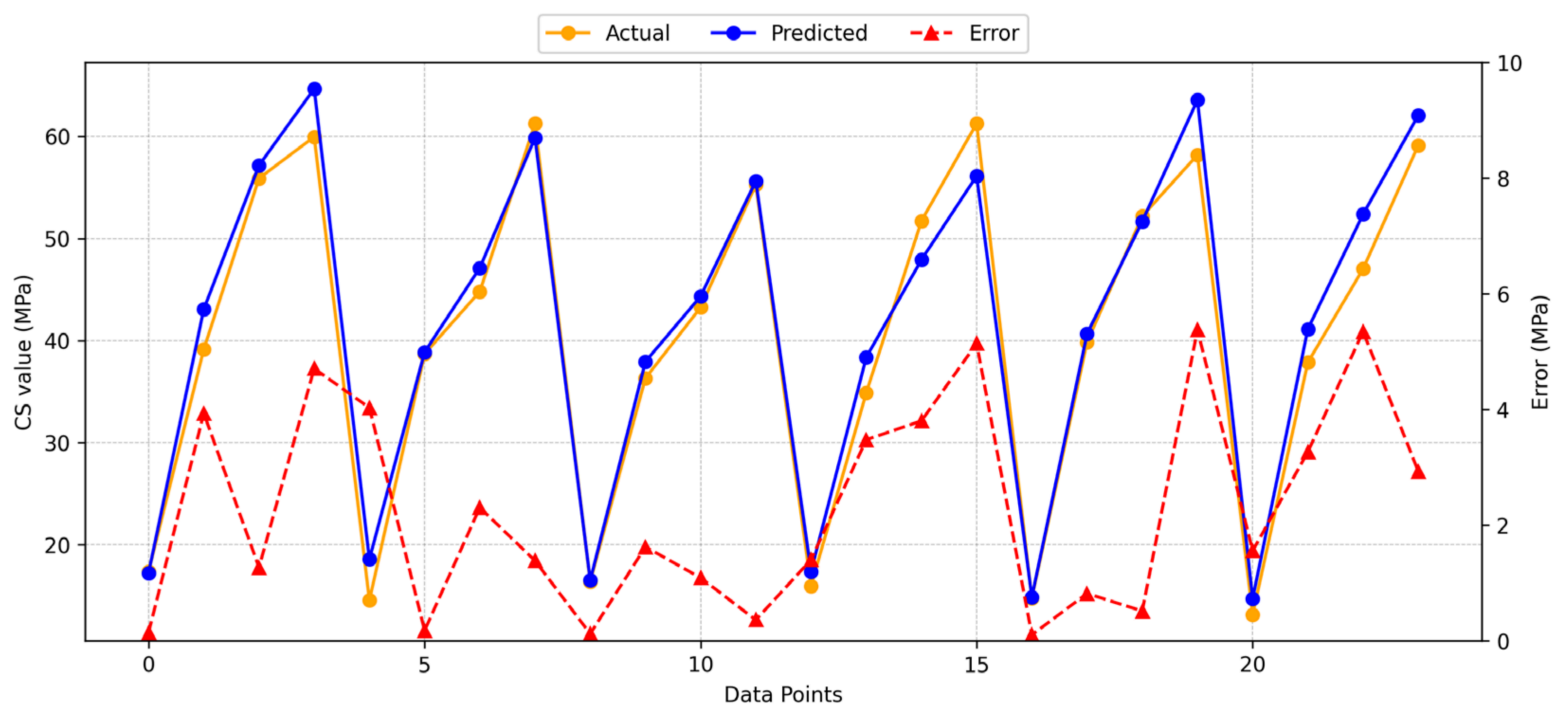
GB model, model estimation, and distribution of accurate and absolute error values for CS.

Figure 7 shows the results obtained from applying the GB model for predicting CS. The GB model demonstrated strong prediction performance with limited variability between the actual and predicted values. The R^2^ score of 0.9692 confirms the satisfactory agreement between the experimental and predicted results. Figure 7 presents the distributions of predicted, actual, and absolute error values for the GB model. The highest observed error value was 5.38 MPa, while the average error value was 2.29 MPa. The obtained results indicate that the GB model produced dependable predictions for CS, although its performance was slightly lower than that of the RF and AB models.

#### 3.2.4. Multilayer Perceptron Model

**Figure 8:**
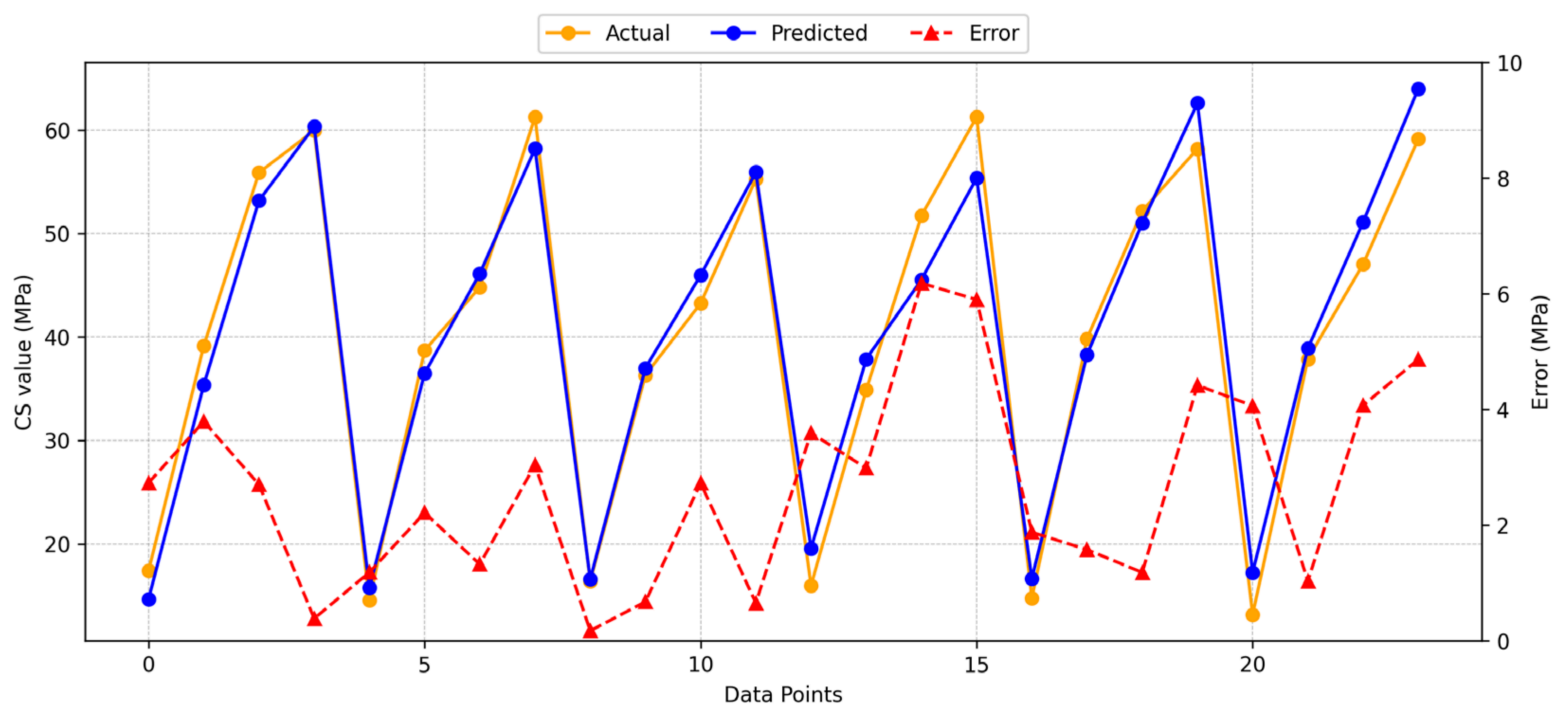
MLP model, model estimation, and distribution of accurate and absolute error values for CS.

Figure 8 presents the results obtained from applying the MLP model for predicting CS. Compared with the ensemble-based models, the MLP model showed lower prediction accuracy and higher variability between the experimental and predicted results. The R^2^ score of 0.9642 indicates acceptable but comparatively weaker agreement between the actual and predicted values. Figure 8 also illustrates the distributions of predicted, actual, and absolute error values for the MLP model. The highest observed error value was 6.18 MPa, while the average error value was 2.64 MPa. The error distribution reveals that the MLP model yielded less stable predictions than the RF, AB, and GB models for CS estimation.

### 3.3. Flow Value Prediction

#### 3.3.1. Random Forest Model

**Figure 9:**
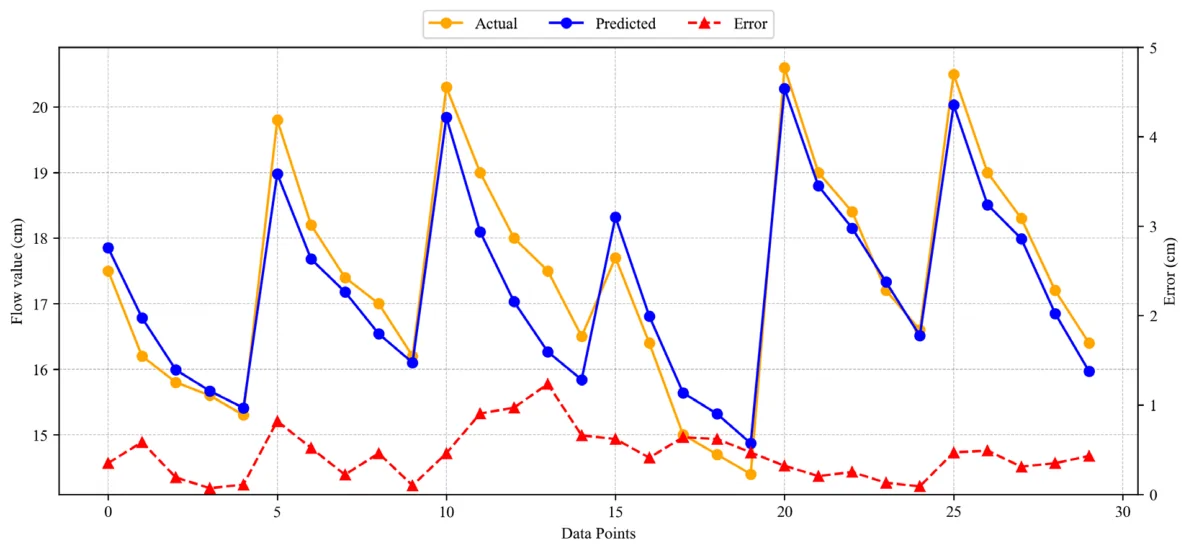
RF model, model estimation, and distribution of accurate and absolute error values for FV.

Figure 9 presents the results obtained from applying the RF model for predicting FV. The RF model demonstrated satisfactory predictive capability with relatively low deviations between the experimental and predicted values. The R^2^ score of 0.9002 indicates good agreement between the actual and predicted results. Figure 9 also illustrates the distribution of predicted, actual, and absolute error values for the RF model. The maximum observed error was 1.24 cm, while the average error value was 0.45 cm. These findings indicate that the RF model provided reliable predictions for FV estimation.

#### 3.3.2. Adaboost Model

**Figure 10:**
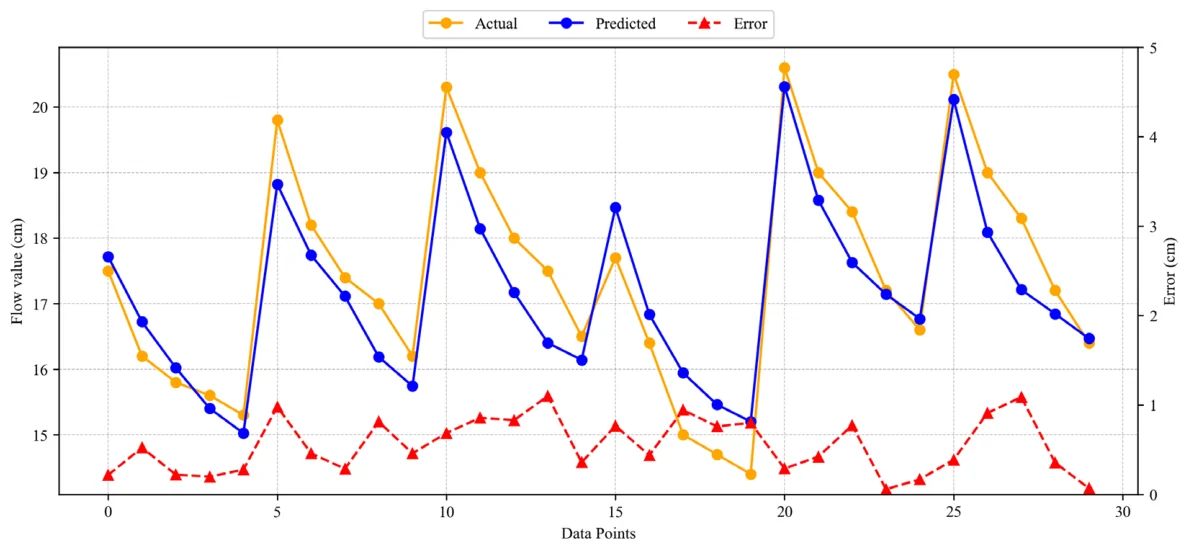
AB model, model prediction, and distribution of accurate and absolute error values for FV.

Figure 10 presents the FV prediction results obtained using the AB model. The AB model achieved moderate prediction performance with an R^2^ value of 0.8573, indicating acceptable agreement between the experimental and predicted values. Compared with the RF and MLP models, the deviations between the actual and predicted values were relatively higher. Figure 10 also shows the distribution of predicted, actual, and absolute error values for the AB model. The maximum observed error was 1.10 cm, while the average error value was 0.55 cm. Overall, the AB model produced acceptable but comparatively less accurate FV predictions.

#### 3.3.3. Gradient Boosting Model

**Figure 11:**
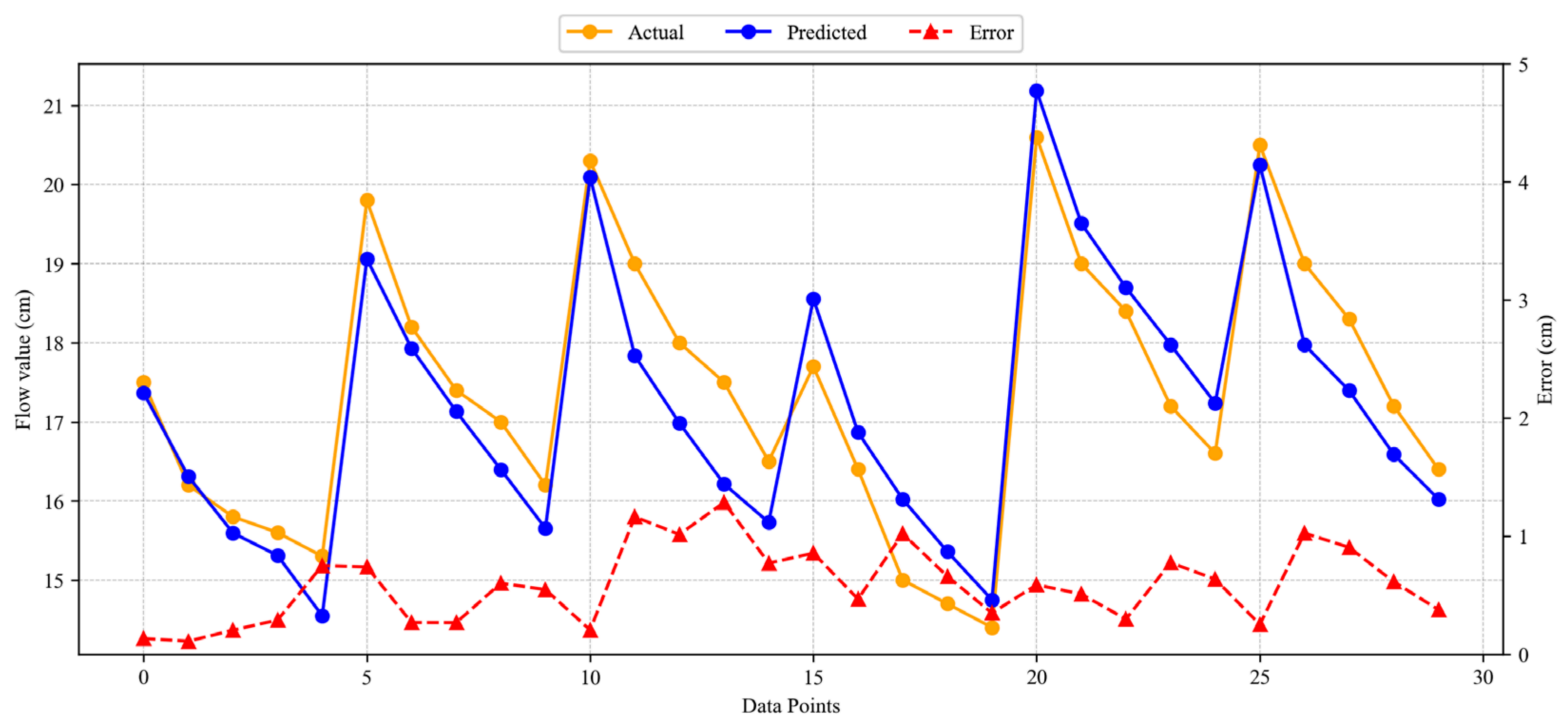
GB model, model estimation, and distribution of accurate and absolute error values for FV.

Figure 11 shows the results obtained from applying the GB model for predicting FV. The GB model demonstrated comparatively lower prediction performance than the RF and MLP models and showed relatively higher variability between the actual and predicted values. The R^2^ score of 0.8387 indicates moderate agreement between the experimental and predicted results. Figure 11 presents the distribution of predicted, actual, and absolute error values for the GB model. The highest observed error value was 1.29 cm, while the average error value was 0.59 cm. The obtained results suggest that the GB model yielded less accurate FV predictions compared to the other evaluated models.

#### 3.3.4. Multilayer Perceptron Model

**Figure 12:**
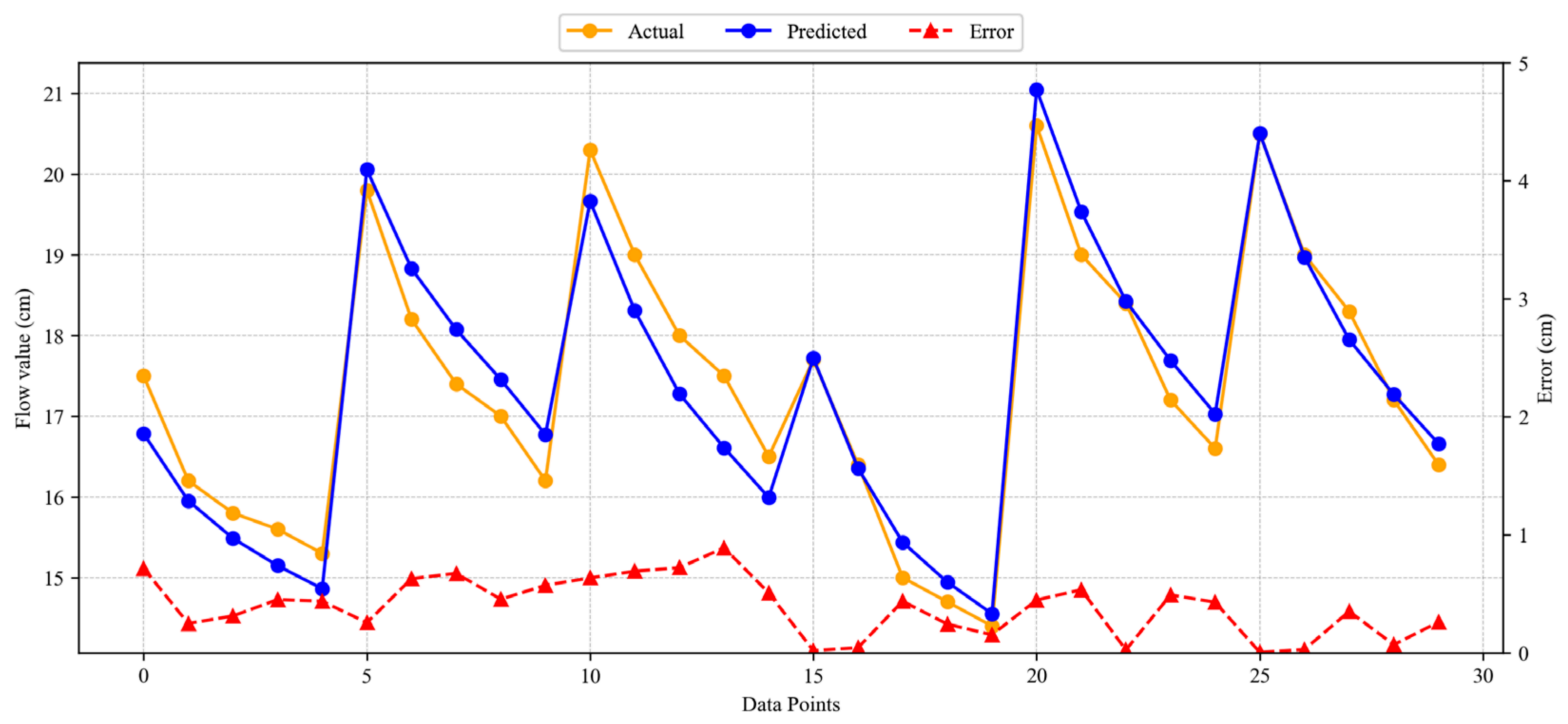
MLP model, model estimation, and distribution of accurate and absolute error values for FV.

Figure 12 presents the results obtained from applying the MLP model for predicting FV. Among the evaluated machine learning models, the MLP model achieved the best overall prediction performance and exhibited lower variability between the actual and predicted values. The R^2^ score of 0.9238 demonstrates good agreement between the experimental and predicted results. Figure 12 also shows the distribution of predicted, actual, and absolute error values for the MLP model. The maximum observed error was 0.89 cm, while the average error value was 0.39 cm. The error distribution indicates that the MLP model provided more consistent FV predictions compared to the RF, AB, and GB models.

### 3.4. Comparing Models Through Statistical Performance Metrics

The MAE, RMSE, MAPE, and R^2^ values obtained from the statistical evaluation of the developed machine learning models are presented in Table 10. In CS prediction, the MAE values were 2.287 MPa for the GB model, 1.911 MPa for the AB model, 1.870 MPa for the RF model, and 2.640 MPa for the MLP model. The MAPE values were 6.22% for the GB model, 5.60% for the AB model, 5.25% for the RF model, and 8.03% for the MLP model. The RMSE values were 2.900 MPa for the GB model, 2.325 MPa for the AB model, 2.280 MPa for the RF model, and 3.126 MPa for the MLP model. The obtained R^2^ values were 0.9692 for GB, 0.9802 for AB, 0.9809 for RF, and 0.9642 for MLP. Among the evaluated models, the RF model achieved the highest R^2^ value together with the lowest overall error metrics, indicating that RF provided the best overall prediction performance for CS estimation.

In FV prediction, the MAE values were 0.590 cm for the GB model, 0.550 cm for the AB model, 0.449 cm for the RF model, and 0.391 cm for the MLP model. The MAPE values were 3.41% for the GB model, 3.16% for the AB model, 2.58% for the RF model, and 2.26% for the MLP model. The RMSE values were 0.670 cm for the GB model, 0.630 cm for the AB model, 0.527 cm for the RF model, and 0.460 cm for the MLP model. In FV prediction, the highest R^2^ value and the lowest MAE, MAPE, and RMSE values were obtained using the MLP model, indicating that MLP achieved the best overall prediction performance among the evaluated models.

**Figure 13:**
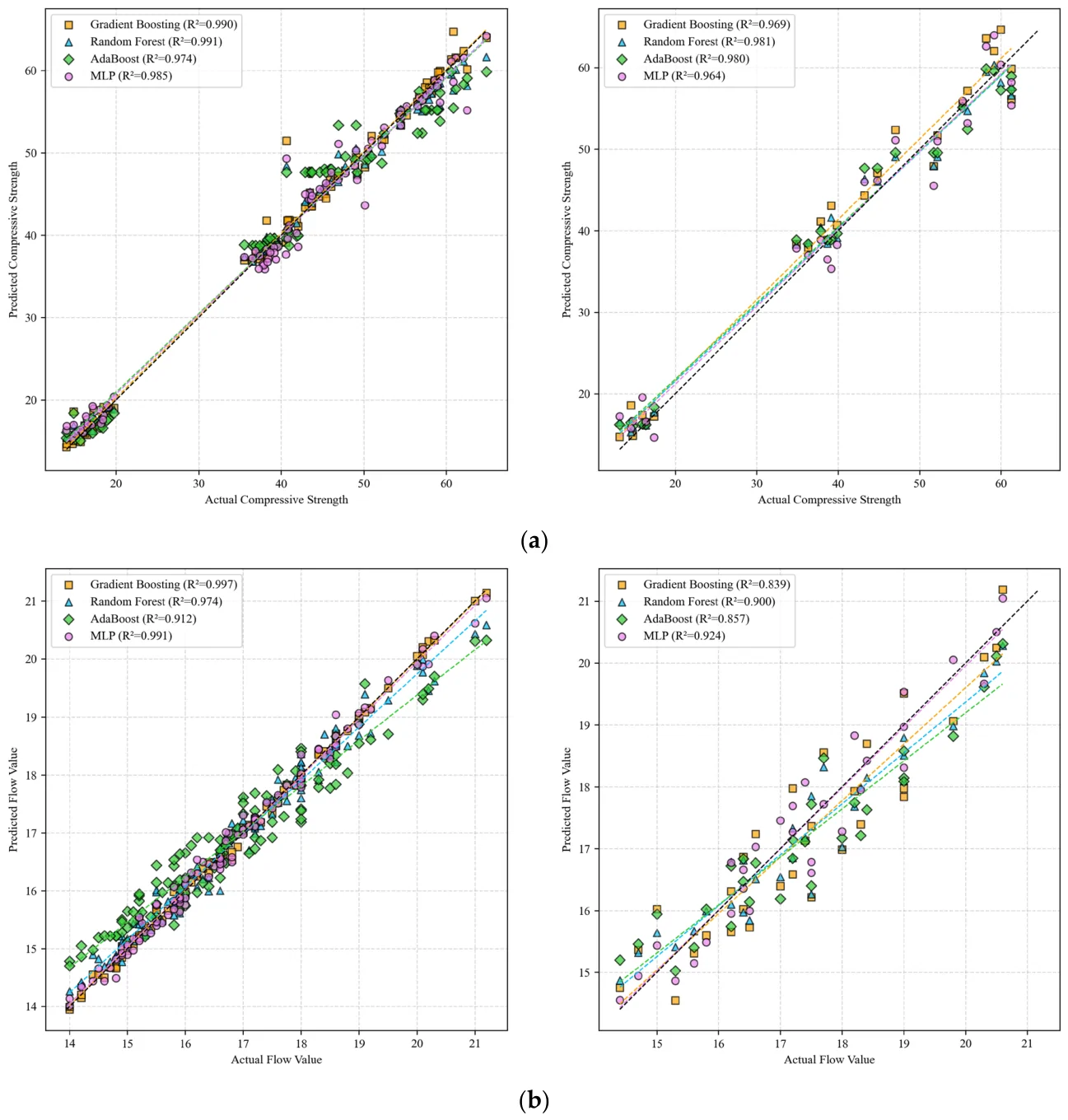
Correlation between actual and predicted values of (**a**) CS and (**b**) FV for training and test.

Figure 13 illustrates the correlation between actual and predicted values for both the training and test datasets. These plots, along with the corresponding R^2^ values, provide a visual comparison of the predictive accuracy of each machine learning model.

Figure 13a presents the results for compressive strength (CS), while Figure 13b displays the outcomes for flow value (FV) predictions.

The fact that the R^2^ values obtained from the training and test sets are highly similar indicates that the models did not suffer from overfitting and possess strong generalization capability. When these results are also considered alongside the Group k-fold cross-validation performance, it further confirms that the models are not overfitted and demonstrate consistent predictive ability across different data partitions.

**Figure 14:**
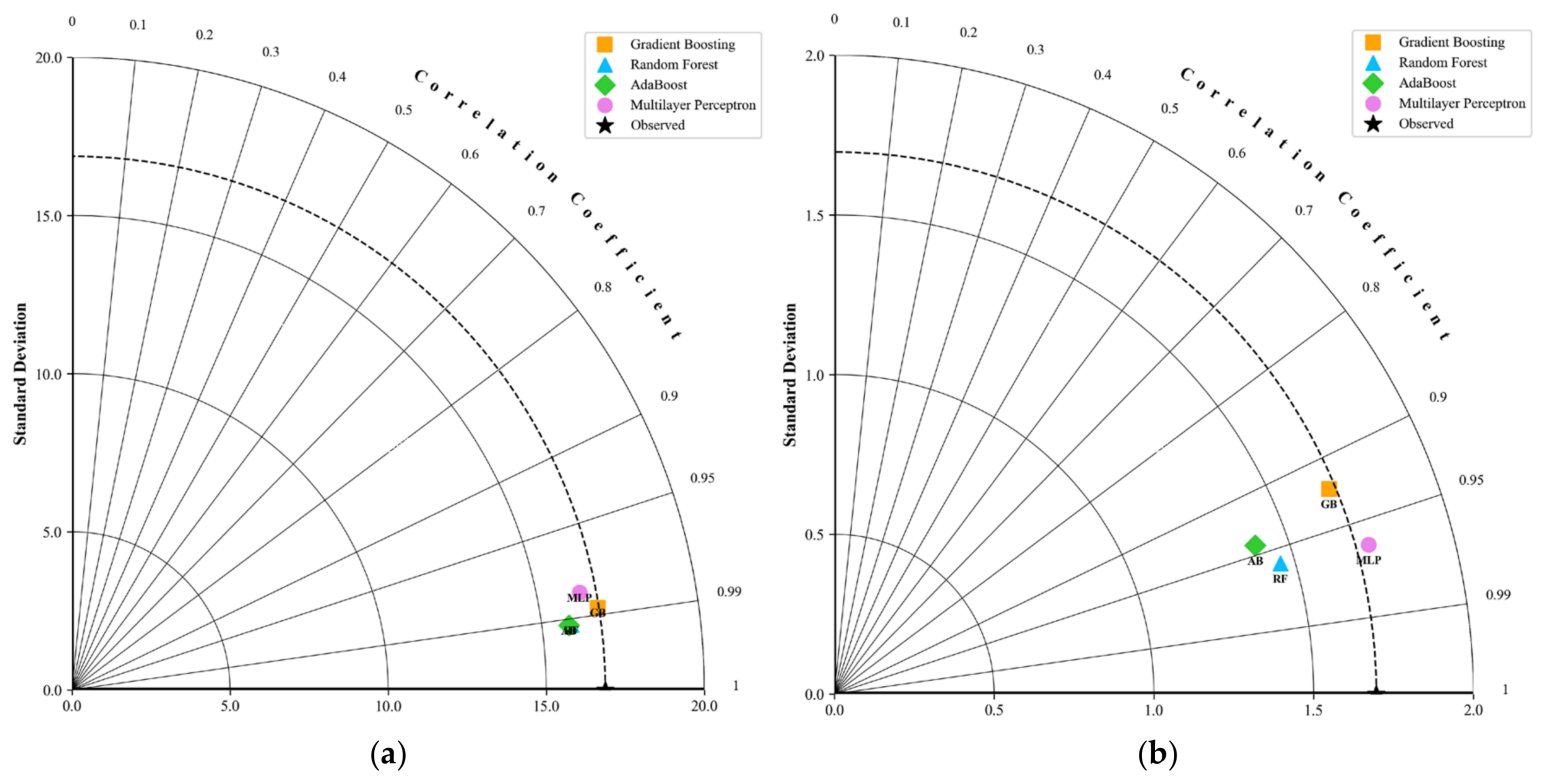
Taylor diagrams for (**a**) CS and (**b**) FV.

The performance of machine learning models was evaluated using Taylor diagrams based on the correlation coefficient (r) and standard deviation values. Results for CS are presented in Figure 14a, while results for FV are shown in Figure 14b.

The correlation coefficients for CS were 0.988 for GB, 0.992 for RF, 0.992 for AB, and 0.982 for MLP, indicating that the RF and AB models best captured the relationship between the observed and predicted values. The standard deviation values were 16.83 for GB, 15.93 for RF, 15.85 for AB, and 16.35 for MLP, with only minor differences observed among the models.

For FV, the correlation coefficients were 0.924 for GB, 0.960 for RF, 0.943 for AB, and 0.963 for MLP, indicating that the MLP model captured the strongest relationship between the observed and predicted values. The standard deviation values were 1.68 for GB, 1.45 for RF, 1.40 for AB, and 1.74 for MLP. Overall, the RF and MLP models demonstrated relatively stronger correlations and prediction performance, whereas the AB model exhibited the lowest standard deviation among the evaluated models.

**Figure 15:**
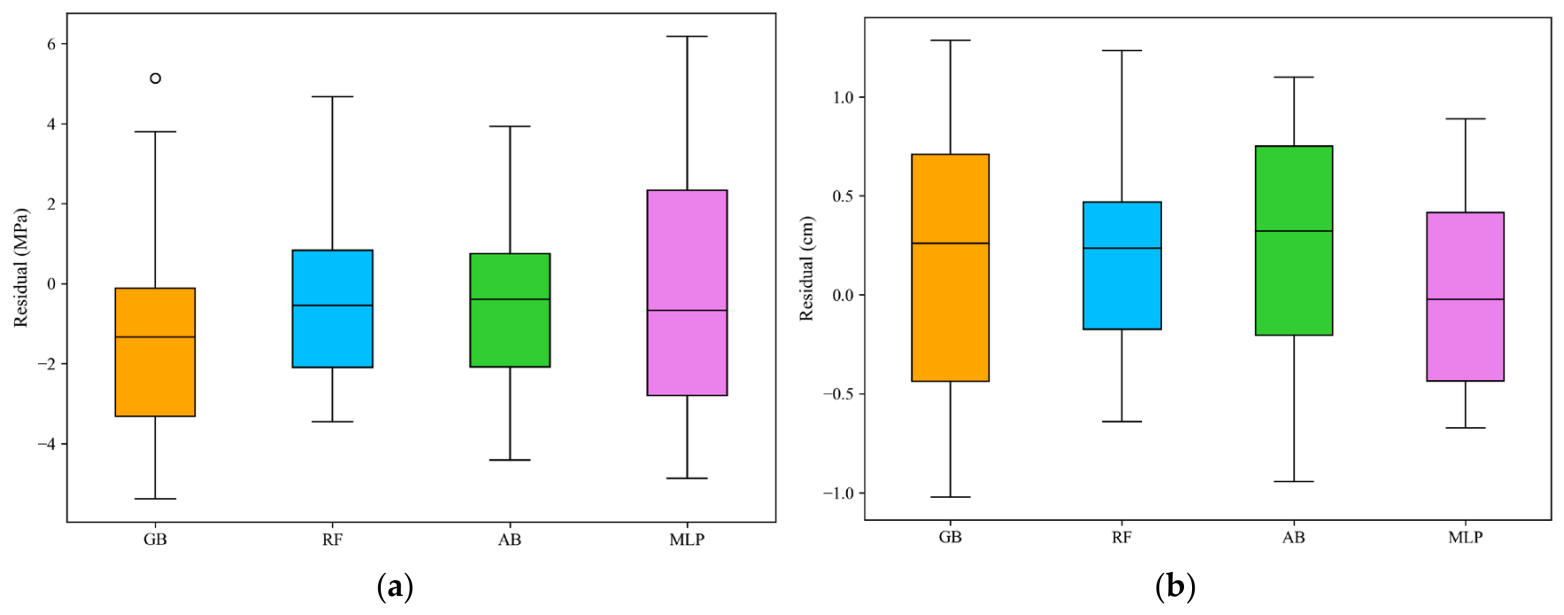
Residual plots for (**a**) CS and (**b**) FV.

Figure 15a and Figure 15b illustrate the residual distributions of the models for compressive strength (CS) and flow value (FV) estimations, respectively.

For CS prediction, the MLP model exhibited the widest residual range and the highest variability among the evaluated models. In contrast, the RF model showed a narrower and more compact residual distribution, indicating that the prediction errors were more closely concentrated around zero. The GB and AB models also demonstrated relatively balanced residual distributions with moderate variability. Although the MLP model produced acceptable prediction performance, its broader residual spread suggests comparatively lower prediction stability than the ensemble-based models. Overall, the residual distributions indicate that the RF and AB models provided more consistent and reliable predictions for CS estimation. For FV, the RF model exhibited the narrowest residual range, indicating a more compact error distribution. The MLP model, on the other hand, showed residuals that were more densely concentrated around zero. In contrast, the AB model demonstrated a relatively wider residual spread, while the GB model exhibited the widest and most variable residual distribution among the evaluated models. These residual distribution patterns suggest that the RF and MLP models produced more stable and reliable predictions for FV estimation.

### 3.5. SHAP Analysis

#### 3.5.1. Compressive Strength

**Figure 16:**
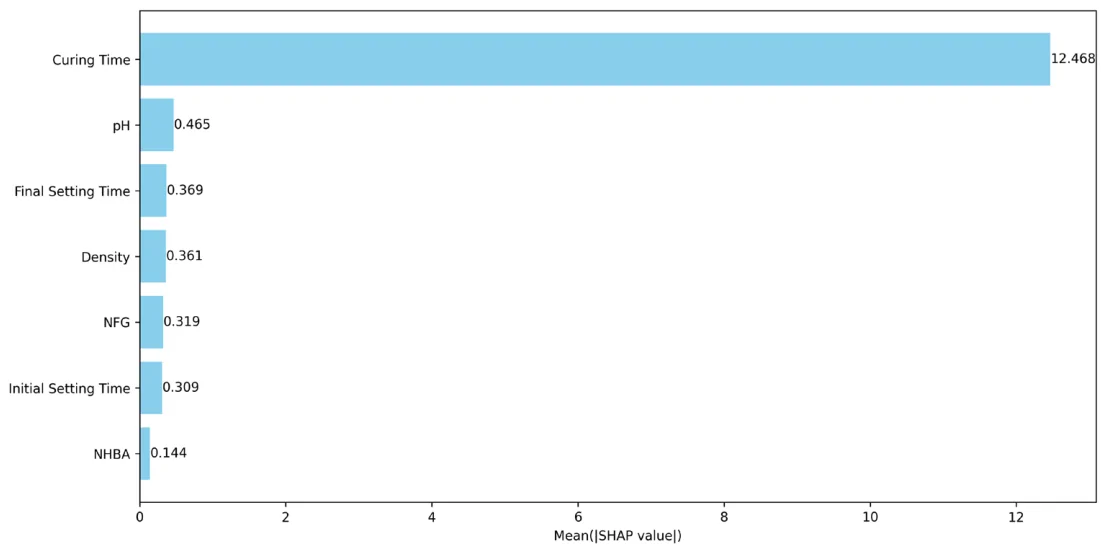
Mean SHAP value-based feature importance analysis for CS value.

Understanding the factors influencing compressive strength in cementitious systems incorporating GAs is essential for optimizing performance. Since the RF model achieved the highest predictive performance among the evaluated models, SHAP analysis was performed using the RF model to provide feature importance interpretations based on the most reliable predictive framework. The SHAP analysis was conducted using the final RF model trained on the complete learning dataset. In this context, a SHAP analysis based on the RF model was conducted to quantitatively evaluate the relative importance of each input feature on compressive strength. As shown in Figure 16, curing time exhibited the highest SHAP importance among the investigated variables. This observation is consistent with the well-established influence of curing duration on cement hydration and strength development. pH was identified as the second most influential parameter, followed by final setting time, density, NFG, initial setting time, and NHBA. However, the importance values of these variables were substantially lower than that of curing time, indicating relatively limited contributions to the RF model predictions. Overall, the SHAP results suggest that curing time contributes more strongly to compressive strength prediction than the other investigated variables, whereas the effects of the remaining physical and grinding-aid-related parameters are comparatively modest within the developed model.

**Figure 17:**
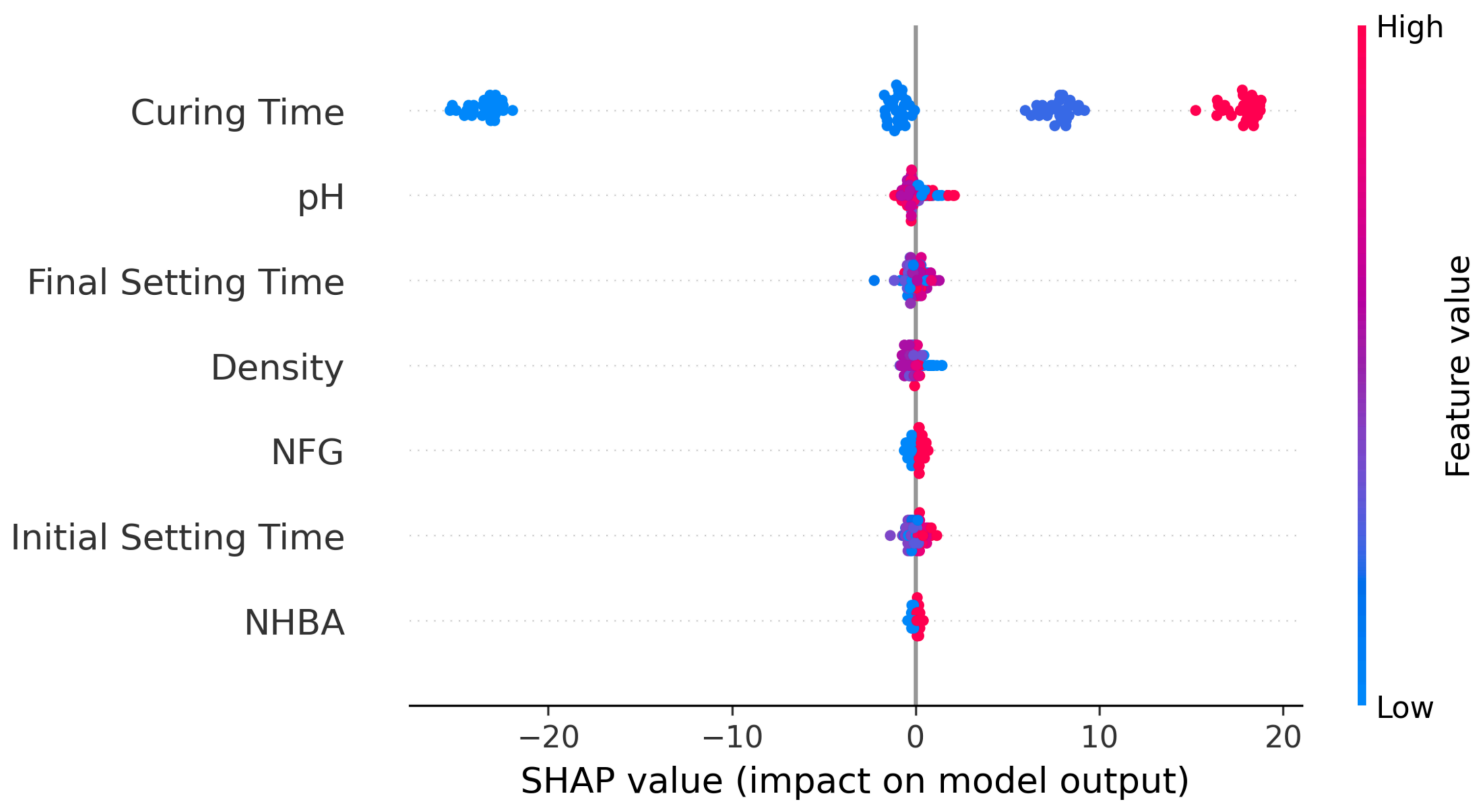
Visualization of feature influence using SHAP summary plot for CS value.

Figure 17 presents the SHAP summary plot for the compressive strength prediction model, illustrating both the magnitude and direction of each input feature’s contribution to the model output. Among all variables, curing time exhibited the greatest influence on the model predictions. Higher curing time values generally produced positive SHAP values and increased the predicted compressive strength, whereas lower curing times were associated with negative SHAP values and lower strength predictions. This pattern indicates that higher curing durations are generally associated with higher predicted compressive strength values within the RF model.

In contrast, the remaining variables exhibited more scattered and overlapping SHAP distributions, making it difficult to identify a clear and consistent trend regarding their influence on compressive strength. Although pH showed the second-highest feature importance, its SHAP values remained concentrated near zero, indicating a substantially weaker influence compared with curing time. Similarly, final setting time, density, NFG, initial setting time, and NHBA displayed narrow SHAP distributions clustered around the origin, suggesting only minor contributions to the model predictions.

Overall, the SHAP analysis performed using the best-performing RF model indicates that curing time is the most influential predictor within the developed model. Nevertheless, the prominence of curing time is expected considering the fundamental role of hydration duration in cementitious materials. Therefore, the SHAP results should primarily be interpreted as an explanation of model behavior rather than evidence of a novel mechanistic finding.

#### 3.5.2. Flow Value

Understanding the factors influencing the flow value in cementitious systems modified with grinding aids (GAs) is important for improving flow performance and overall material behavior. Since the MLP model achieved the highest predictive performance for flow value prediction, SHAP analysis was performed using the MLP model to provide feature importance interpretations based on the most reliable predictive framework. The SHAP analysis was conducted using the final MLP model trained on the complete learning dataset. Accordingly, a SHAP analysis based on the MLP model was conducted to quantitatively evaluate the relative importance of each input feature on the flow value.

**Figure 18:**
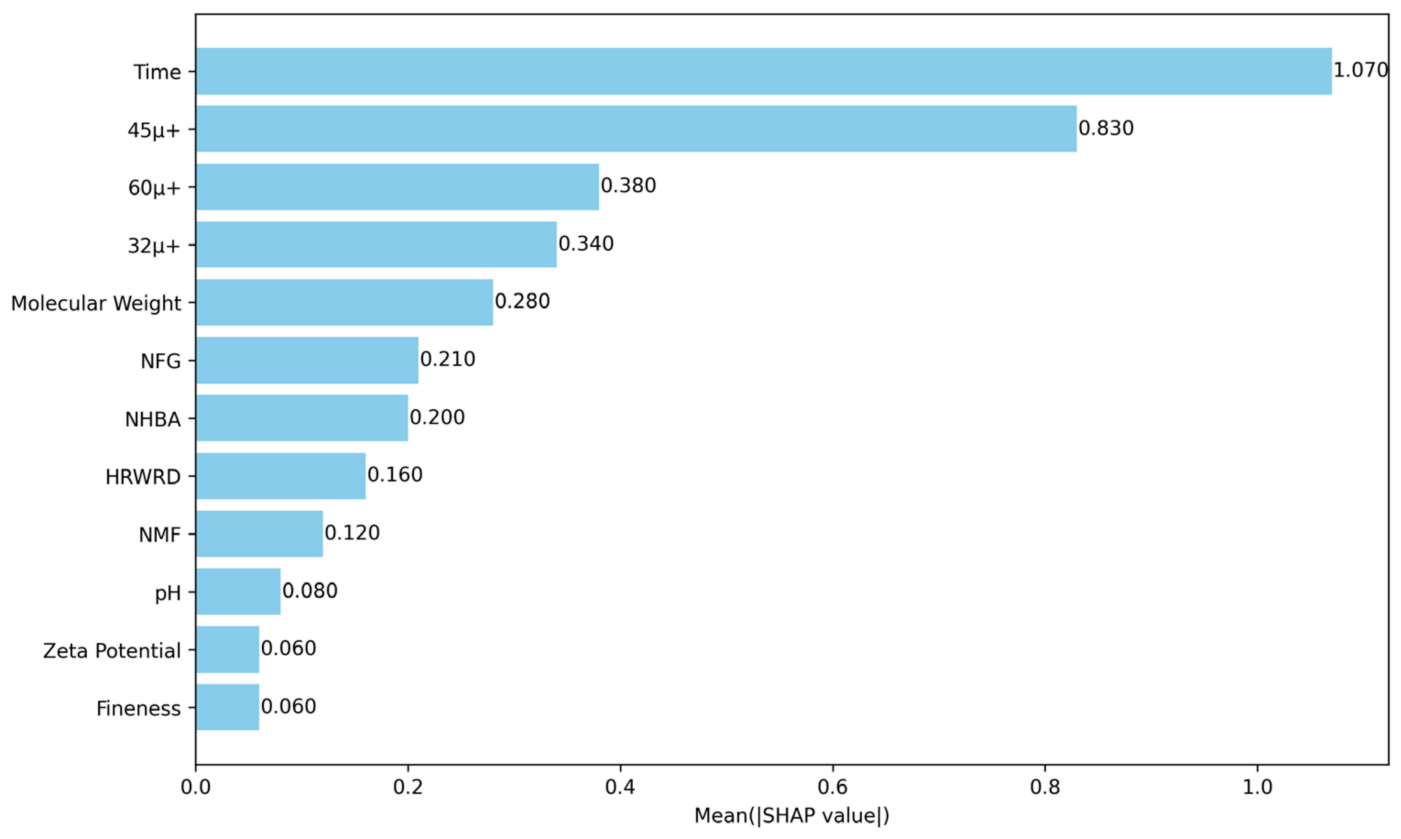
Mean SHAP value-based feature importance analysis for FV.

As shown in Figure 18, time was identified as the most influential parameter affecting the flow value, followed by the 45μ+ particle fraction. The importance values of these two variables were noticeably higher than those of the remaining features, indicating that the MLP model relied primarily on temporal behavior and coarse particle size distribution during prediction. The 60μ+ and 32μ+ particle fractions were also found to have notable contributions, followed by molecular weight, NFG, NHBA, and HRWRD. In contrast, NMF, pH, zeta potential, and fineness exhibited relatively low SHAP importance values. Overall, the SHAP results suggest that time and particle size distribution characteristics contribute more strongly to flow value prediction than the remaining investigated variables within the developed model.

**Figure 19:**
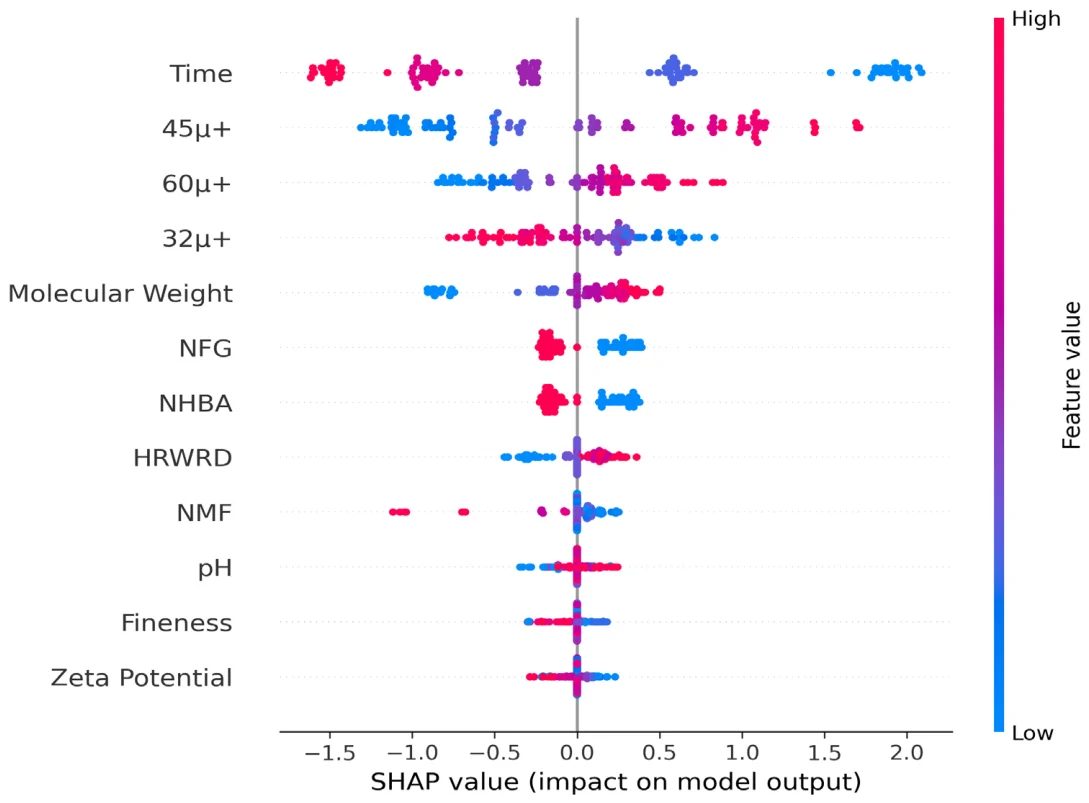
Visualization of feature influence using SHAP summary plot for FV.

Figure 19 provides a detailed overview of the influence of each input feature on the flow value, highlighting both positive and negative contributions.

Figure 19 presents the SHAP summary plot for the flow value prediction model, showing the magnitude and direction of each input parameter’s contribution to the model output. Among all variables, time exhibited the greatest influence on the model predictions. Lower time values generally contributed positively to the predicted flow value, whereas higher time values were associated with negative SHAP values, indicating a reduction in flowability over time. This pattern is consistent with the expected loss of workability and flowability as elapsed time increases in cementitious systems.

The 45μ+ particle fraction also exerted a noticeable influence on the model output, with higher values generally increasing the predicted flow value and lower values generally decreasing it. Similarly, the 60μ+ particle fraction showed a predominantly positive contribution at higher values, whereas the 32μ+ fraction exhibited an opposite tendency, with higher values generally associated with lower SHAP values. Molecular weight showed a moderate positive relationship with the predicted flow value, while NFG and NHBA displayed relatively weak but distinguishable effects on the model output.

In contrast, HRWRD, NMF, pH, fineness, and zeta potential exhibited SHAP values concentrated near zero, indicating comparatively limited contributions to the model predictions. Overall, the SHAP analysis performed using the best-performing MLP model indicates that time and particle size distribution characteristics are the most influential predictors within the developed model. Nevertheless, the prominence of time is expected considering the well-known time-dependent rheological behavior of cementitious materials. Therefore, the SHAP results should primarily be interpreted as an explanation of model behavior rather than evidence of a novel mechanistic finding.

## 4. Conclusions

This study investigated the application of four distinct machine learning algorithms to predict the dispersion and strength characteristics of cementitious mixtures incorporating cements produced with varying GA types and dosages. The predictive capabilities of each model were evaluated by comparing its outputs to experimentally determined results. The key findings of this research are summarized below:

Upon evaluation of the experimental results, it was observed that, in terms of flow and consistency retention, the M-TEA-1 and M-TEA-2 mixtures exhibited better performance than those produced with other GA compounds. In terms of strength, mixtures containing DEIPA, M-TEA-1, and M-TEA-2 showed the best long-term compressive strength. This indicates that these GAs and the TEA modification have yielded successful results.

The computational results showed that RF exhibited the best overall performance for CS prediction, while MLP provided comparatively better results for FV prediction. Additionally, for the specific predicted values, all four models produced satisfactory results for CS predictions, with RF achieving the highest accuracy (R^2^ = 0.9809), followed closely by AB, GB, and MLP. On the other hand, for FV predictions, MLP achieved the highest R^2^ value (0.9238) and the lowest error metrics, while RF and AB also demonstrated satisfactory predictive capability, whereas GB’s performance was comparatively lower.

As a result, the computational experiments indicate that all four machine learning algorithms considered in this study can be effectively used for CS prediction, whereas MLP, RF, and GB models demonstrated comparatively better performance for FV prediction.

Additionally, SHAP analysis provided valuable insights into the behavior of the best-performing predictive models (RF for CS and MLP for FV). The results indicated that curing time contributed most strongly to the RF model predictions for compressive strength, while time and particle size distribution variables showed relatively higher importance in the MLP model developed for flow value prediction. These explainability results should be interpreted as model-based importance assessments rather than direct evidence of mechanistic relationships. Nevertheless, they improve model interpretability and provide useful guidance for the optimization of cementitious mixtures containing grinding aids.

However, it should be noted that the present study is based on a relatively limited experimental dataset and does not include external validation using independent industrial-scale data. In addition, the experimental program provides limited information regarding data variability, as compressive strength values were reported as averages without standard deviations or confidence intervals, and flow measurements were generally performed once per mixture. These factors restrict a comprehensive statistical assessment of experimental uncertainty and should be considered when interpreting the predictive performance of the developed models. Therefore, future work will focus on validating the proposed models using larger, independently generated datasets with repeated measurements to improve statistical robustness, generalization, and reliability. Future studies may integrate molecular simulation descriptors to better capture GA–PCE compatibility effects in data-driven prediction frameworks.

## Figures and Tables

**Table 1:** Some properties of the clinker and gypsum used in this study.

	SiO_2_	Al_2_O_3_	Fe_2_O_3_	CaO	MgO	SO_3_	Na_2_O_eq_	Cl	Combined Water (T < 230°)	Other. %	C_3_S	C_2_S	C_3_A	C_4_AF	Loss of Ignition
Clinker	21.52	5.43	3.31	65.38	1.04	0.38	0.83	0.01	-	1.58	56.51	19.06	8.79	10.07	0.52
Gypsum	4.98	1.21	0.83	28.94	0.83	39.67	0.37	-	18.93	4.24					

**Table 2:** Some physical and chemical properties of the chemicals used.

Type of Chemical	Molecular Formula	Molecular Weight (g/mol)	Density (g/cm ^3^)	pH 25 °C
TEA	C_6_H_15_NO_3_	149.19	1.13	10.5
TIPA	C_9_H_21_NO_3_	191.27	1.12	10.8
DEIPA	C_7_H_17_NO_3_	163.14	1.08	9.8
EG	C_2_H_6_O_2_	62.07	1.11	7.0
DEG	C_4_H_10_O_3_	106.12	1.11	7.2
	**Backbone length (g/mol)**	**Side chain length (g/mol)**	**Molecular weight (g/mol)**	**Side c./backbone ratio**
PCE type GA	4000	2400	37,000	14

**Table 3:** PCE requirement for the desired flow and time-dependent slump values of mortar mixtures with a constant PCE amount [40].

	PCE Requirement for the Desired Flow		Time-Dependent Flow Values of Mortar Mixtures with Constant PCE Amount (%0.36)				
Mixtures	PCE Dosage (%) *	Desired Flow Value (cm)	0th min (cm)	15th min. (cm)	30th min. (cm)	45th min. (cm)	60th min. (cm)
Control	0.340	19.3	20.7	18.25	17.5	17.0	16.25
0.025 TEA	0.360	19.0	19.0	17.75	17.0	16.5	16.0
0.05 TEA	0.365	18.8	18.5	16.75	16.0	15.25	14.8
0.075 TEA	0.370	18.7	18.0	16.65	15.9	14.9	14.4
0.1 TEA	0.380	18.8	17.0	15.5	15.0	14.8	14.0
0.025 TIPA	0.350	19.2	20.2	18.6	18.0	17.2	16.6
0.05 TIPA	0.360	18.6	18.6	17.2	16.4	15.9	15.1
0.075 TIPA	0.372	18.3	17.0	16.6	15.8	15.5	15.0
0.1 TIPA	0.368	18.2	17.5	16.2	15.8	15.6	15.3
0.025 DEIPA	0.350	19.1	20.3	19.2	18.5	17.7	16.7
0.05 DEIPA	0.356	19.4	19.8	18.2	17.4	17.0	16.2
0.075 DEIPA	0.362	18.6	18.3	17.2	16.2	15.8	15.0
0.1 DEIPA	0.368	18.2	17.6	16.0	15.6	15.0	14.2
0.025 DEG	0.350	19.2	20.3	19.0	18.0	17.5	16.5
0.05 DEG	0.352	19.2	20.1	18.8	18.0	17.1	16.6
0.075 DEG	0.352	19.1	20.1	18.3	17.5	16.7	16.4
0.1 DEG	0.360	19.0	19.0	17.5	16.3	16.0	15.3
0.025 EG	0.362	18.7	18.4	16.9	15.8	15.5	14.9
0.05 EG	0.366	18.5	17.9	16.6	15.9	15.4	14.5
0.075 EG	0.368	18.4	17.4	16.1	15.7	15.1	14.2
0.1 EG	0.372	18.1	17.0	15.8	15.2	14.7	14.0
0.025 M-TEA-1	0.345	19.1	20.0	18.0	17.2	16.2	15.9
0.05 M-TEA-1	0.360	19.1	19.1	17.6	16.8	15.5	15.2
0.075 M-TEA-1	0.365	19.2	18.0	16.8	15.2	14.9	14.6
0.1 M-TEA-1	0.370	18.7	17.7	16.4	15.0	14.7	14.4
0.025 M-TEA-2	0.335	19.0	21.2	19.5	18.6	18.0	17.2
0.05 M-TEA-2	0.340	19.1	20.6	19.0	18.4	17.2	16.6
0.075 M-TEA-2	0.335	19.2	21.0	18.6	18.0	17.3	16.6
0.1 M-TEA-2	0.340	18.9	20.5	19.0	18.3	17.2	16.4

* PCE requirement (by weight of cement%) for desired flow value (cm).

**Table 4:** The compressive strength results of the mixtures.

Mixtures	Compressive Strength (MPa)							
	1-Day	1-Day SD *	3-Day	3-Day SD	7-Day	7-Day SD	28-Day	28-Day SD
Control	16.53	0.82	38.91	1.33	46.73	1.54	49.66	1.63
0.025 TEA	16.17	0.84	38.42	1.41	45.92	1.48	54.46	1.71
0.05 TEA	18.16	0.87	36.49	1.33	45.24	1.51	49.05	1.53
0.075 TEA	18.46	0.93	39.60	1.36	46.06	1.50	46.94	1.54
0.1 TEA	19.75	0.99	40.94	1.43	52.18	1.70	59.21	1.81
0.025 TIPA	16.45	0.79	40.77	1.53	52.46	1.62	58.57	1.72
0.05 TIPA	17.96	0.81	40.78	1.42	57.04	1.74	62.09	1.81
0.075 TIPA	16.35	0.87	41.86	1.44	56.48	1.71	61.14	1.84
0.1 TIPA	17.39	0.93	39.14	1.41	55.88	1.78	59.95	1.83
0.025 DEIPA	13.98	0.99	39.35	1.50	46.90	1.56	59.20	1.81
0.05 DEIPA	14.56	0.77	38.69	1.36	44.80	1.46	61.24	1.80
0.075 DEIPA	14.85	0.81	38.22	1.41	40.64	1.40	60.86	1.85
0.1 DEIPA	14.71	0.87	38.01	1.42	43.46	1.48	58.19	1.74
0.025 DEG	16.40	0.93	36.31	1.29	43.24	1.45	55.26	1.69
0.05 DEG	15.99	0.99	37.75	1.40	44.68	1.48	55.18	1.74
0.075 DEG	16.22	0.75	37.88	1.35	43.31	1.42	54.46	1.71
0.1 DEG	15.06	0.81	37.25	1.37	43.60	1.42	54.37	1.73
0.025 EG	19.08	0.87	39.10	1.43	45.40	1.53	57.62	1.78
0.05 EG	17.03	0.93	35.52	1.31	49.19	1.62	59.00	1.80
0.075 EG	17.27	0.99	40.54	1.39	42.90	1.45	58.86	1.74
0.1 EG	18.90	0.75	39.18	1.47	43.72	1.44	57.42	1.74
0.025 M-TEA-1	18.38	0.81	36.88	1.32	48.92	1.56	57.90	1.74
0.05 M-TEA-1	17.16	0.87	38.31	1.34	50.11	1.63	62.51	1.83
0.075 M-TEA-1	16.52	0.93	37.41	1.29	47.74	1.59	56.69	1.79
0.1 M-TEA-1	15.97	0.99	34.87	1.29	51.71	1.68	61.26	1.82
0.025 M-TEA-2	15.67	0.75	38.63	1.42	50.49	1.64	60.60	1.87
0.05 M-TEA-2	14.77	0.81	39.84	1.36	52.18	1.66	58.18	1.79
0.075 M-TEA-2	14.00	0.87	42.05	1.51	50.93	1.58	64.85	1.95
0.1 M-TEA-2	13.17	0.93	37.86	1.33	47.02	1.50	59.12	1.79

* SD: Standard deviation represents the standard deviation calculated based on the testing of three replicate specimens for each mixture (n = 3).

**Table 5:** Statistical characteristics of the dataset.

Parameter	Input Variables																Output	
	F	32μ+	45μ+	60μ+	ZP	HRWRD	MW	NMF	Density	pH	NHBA	NFG	IST	FST	*CT*	*T*	CS	FV
						(%)											(MPa)	(cm)
Mean	4114.82	19.5	6.11	0.68	−5.72	0.06	159.26	1.72	1.12	7.8	3.57	2.57	203.25	311.79	*9.75*	*30*	40.25	16.93
Standard Error	4.46	0.53	0.25	0.02	0.25	0	5.29	0.12	0.01	0.26	0.05	0.05	2.77	4.39	*1.02*	*1.8*	1.49	0.14
Median	4105	20	5.9	0.7	−5.58	0.06	163.2	1.43	1.12	8.2	4	3	196	316	*5*	*30*	41.96	16.73
Mode	4100	21	9	0.9	−9.41	0.03	62.07	0.39	1.02	2.63	4	3	185	235	*1*	*0*	52.18	18
Standard Deviation	47.22	5.62	2.65	0.24	2.61	0.03	56	1.22	0.05	2.74	0.5	0.5	29.36	46.49	*10.8*	*21.29*	15.75	1.66
Range	180	18.7	9.1	0.9	8.51	0.08	174.93	5.44	0.18	8.17	1	1	110	161	*27*	*60*	51.68	7.2
Minimum	4020	9.5	2.1	0.2	−9.41	0.03	62.07	0.39	1.02	2.63	3	2	156	235	*1*	*0*	13.17	14
Maximum	4200	28.2	11.2	1.1	−0.9	0.1	237	5.83	1.2	10.8	4	3	266	396	*28*	*60*	64.85	21.2

**F:** Fineness. **ZP:** Zeta Potential. **MW:** Molecular Weight. **NMF:** Number of Molecules per Fineness. **NHBA:** Number of Hydrogen Bond Acceptors. **NFG:** Number of Functional Groups. ***IST:*** Initial Setting Time. **FST:** Final Setting Time. **CT:** Curing Time. **T:** Time. **CS:** Compressive Strength. **FV:** Flow Value.

**Table 6:** Hyperparameter settings for ML models in compressive strength estimation.

Parameter	RF		AB		GB		MLP	
	Range/ Step Size	Optimal. Value	Range/ Step Size	Optimal. Value	Range/ Step Size	Optimal. Value	Range/ Step Size	Optimal. Value
No. of estimators	10–200/50	50	10–200/50	50	10–200/50	10	-	-
Learning rate	-	-	0.01–1.0/0.1	0.3	0.01–1.0/0.1	0.9	-	-
Max. depth	10–20/5	10	-	-	1–5/1	4	-	-
Max. features	0.8–1.0/0.1	0.8	-	-	0.8–1.0/0.1	0.9	-	-
Min. sample leaf	1–4/1	1	-	-	1–4/1	2	-	-
Min. sample split	2–10/2	2	-	-	2–12/2	10	-	-
Hidden layer size	-	-	-	-	-	-	(50,)–(200, 150, 100)	(50,)
Activation Func.	-	-	-	-	-	-	[relu, tanh, logistic]	logistic
Alpha	-	-	-	-	-	-	0.001–0.5	0.5
Optimization alg.	-	-	-	-	-	-	[adam, lbfgs, sgd]	lbfgs
Max. iterations	-	-	-	-	-	-	-	300

**Table 7:** Hyperparameter settings for ML models in the flow value estimation.

Parameter	RF		AB		GB		MLP	
	Range/ Step Size	Opt. Value	Range/ Step Size	Opt. Value	Range/ Step Size	Opt. Value	Range/ Step Size	Opt. Value
No. of estimators	10–200/50	10	10–200/50	100	10–200/50	200	-	-
Learning rate	-	-	0.01–1.0/0.1	0.6	0.01–1.0/0.1	0.4	-	-
Max. depth	10–20/5	15	-	-	1–5/1	2	-	-
Max. features	0.8–1.0/0.1	0.8	-	-	0.8–1.0/0.1	0.8	-	-
Min. sample leaf	1–4/1	1	-	-	1–4/1	4	-	-
Min. sample split	2–10/2	2	-	-	2–12/2	10	-	-
Hidden layer size	-	-	-	-	-	-	(50,)–(200, 150, 100)	(150,)
Activation Func.	-	-	-	-	-	-	[relu, tanh, logistic]	tanh
Alpha	-	-	-	-	-	-	0.001–0.5/log scale	0.5
Optimization alg.	-	-	-	-	-	-	[adam, lbfgs, sgd]	lbfgs
Max. iterations	-	-	-	-	-	-	-	300

**Table 8:** Group K-fold cross-validation results for optimal hyperparameters in compressive strength estimation.

Model	Parameter	K-Fold Number										Avg.
		1	2	3	4	5	6	7	8	9	10	
RF	MAE	1.28	1.54	2.76	2.75	4.27	2.99	2.64	1.21	1.80	3.52	2.48
	MAPE	3.74	4.47	7.77	6.62	11.05	7.84	7.96	2.56	5.12	10.37	6.75
	RMSE	1.80	1.80	3.33	3.26	5.17	3.40	2.90	1.87	2.08	4.61	3.02
	R^2^	0.99	0.99	0.93	0.94	0.87	0.95	0.97	0.99	0.98	0.92	0.95
AB	MAE	2.06	0.98	2.37	2.06	3.02	1.83	2.72	2.40	1.18	2.89	2.15
	MAPE	5.48	3.37	6.25	5.57	7.73	4.27	6.51	5.82	4.81	8.54	5.84
	RMSE	2.92	1.16	2.85	2.97	3.94	2.55	3.18	2.92	1.47	3.65	2.76
	R^2^	0.97	0.99	0.95	0.95	>0.93	0.97	0.96	0.97	0.99	0.95	0.96
GB	MAE	2.31	1.18	2.13	2.13	2.85	1.87	2.20	2.09	0.91	2.76	2.04
	MAPE	7.16	4.07	5.70	6.26	6.39	5.10	5.51	5.09	3.75	7.41	5.64
	RMSE	2.93	1.30	2.57	3.21	4.51	2.51	2.61	2.78	1.05	3.54	2.70
	R^2^	0.97	0.99	0.96	0.94	0.90	0.98	0.97	0.97	1.00	0.95	0.96
MLP	MAE	2.92	1.63	1.49	2.05	3.31	4.34	2.12	3.11	2.44	3.82	2.72
	MAPE	7.04	3.88	3.94	5.05	7.45	12.46	5.84	9.31	8.30	10.04	7.33
	RMSE	3.67	2.20	1.70	3.07	4.76	5.03	2.84	3.91	2.76	4.84	3.48
	R^2^	0.95	0.98	0.98	0.94	0.89	0.90	0.97	0.94	0.97	0.91	0.94

**Table 9:** Group K-fold cross-validation for optimal hyperparameters in flow value estimation.

Model	Parameter	K-Fold Number										Avg.
		1	2	3	4	5	6	7	8	9	10	
RF	MAE	0.65	0.44	0.42	1.03	0.20	0.69	0.91	0.55	0.77	0.79	0.65
	MAPE	3.76	2.57	2.53	6.05	1.24	3.99	5.26	3.22	4.38	4.63	3.76
	RMSE	0.82	0.60	0.54	1.10	0.28	0.79	1.05	0.69	0.84	0.88	0.76
	R^2^	0.77	0.90	0.78	0.66	0.95	0.76	0.64	0.77	0.71	0.69	0.76
AB	MAE	0.82	0.68	0.39	0.90	0.66	0.71	0.75	0.68	0.56	0.88	0.70
	MAPE	4.79	4.01	2.33	5.37	4.23	4.18	4.47	3.91	3.14	5.32	4.18
	RMSE	0.94	0.77	0.48	0.98	0.72	0.79	0.85	0.90	0.64	0.95	0.80
	R^2^	0.69	0.83	0.82	0.73	0.71	0.76	0.77	0.61	0.83	0.65	0.74
GB	MAE	0.84	0.66	0.42	0.69	0.53	0.85	0.85	0.80	0.72	0.71	0.71
	MAPE	4.94	3.87	2.56	4.11	3.40	4.93	5.01	4.60	4.00	4.21	4.16
	RMSE	0.97	0.72	0.50	0.72	0.64	0.93	0.97	0.98	0.81	0.76	0.80
	R^2^	0.67	0.85	0.81	0.85	0.76	0.68	0.69	0.54	0.73	0.77	0.74
MLP	MAE	0.34	0.64	0.44	0.76	0.26	0.37	0.48	0.46	0.54	0.52	0.48
	MAPE	2.16	3.79	2.69	4.43	1.66	2.22	2.79	2.72	2.97	3.00	2.84
	RMSE	0.47	0.71	0.48	0.81	0.29	0.44	0.56	0.55	0.69	0.62	0.56
	R^2^	0.92	0.86	0.82	0.81	0.95	0.93	0.90	0.85	0.80	0.85	0.87

**Table 10:** Performance metrics of the constructed machine learning models.

	Compressive Strength				Flow Value			
Model	R^2^	MAE (MPa)	MAPE (%)	RMSE (MPa)	R^2^	MAE (cm)	MAPE (%)	RMSE (cm)
GB	0.9692	2.287	6.220	2.900	0.8387	0.590	3.410	0.670
AB	0.9802	1.911	5.600	2.325	0.8573	0.550	3.160	0.630
RF	0.9809	1.870	5.250	2.280	0.9002	0.449	2.580	0.527
MLP	0.9642	2.640	8.030	3.126	0.9238	0.391	2.260	0.460

## Data Availability

The original contributions presented in this study are included in the article. Further inquiries can be directed to the corresponding author.
